# Supplementary material for: Global projections of heat exposure of older adults
Source: Nat Commun. 2024 May 14;15:3678. doi: 10.1038/s41467-024-47197-5 (PMC11094092; doi:10.1038/s41467-024-47197-5)
Supplement: Supplementary file 1 — Supplementary Information [file 41467_2024_47197_MOESM1_ESM.pdf]

Aging in a warming world: global projections of heat exposure of older adults

Giacomo Falchetta et al. (2024)

Supplementary Information

Gridded age-stratified population projections comparison

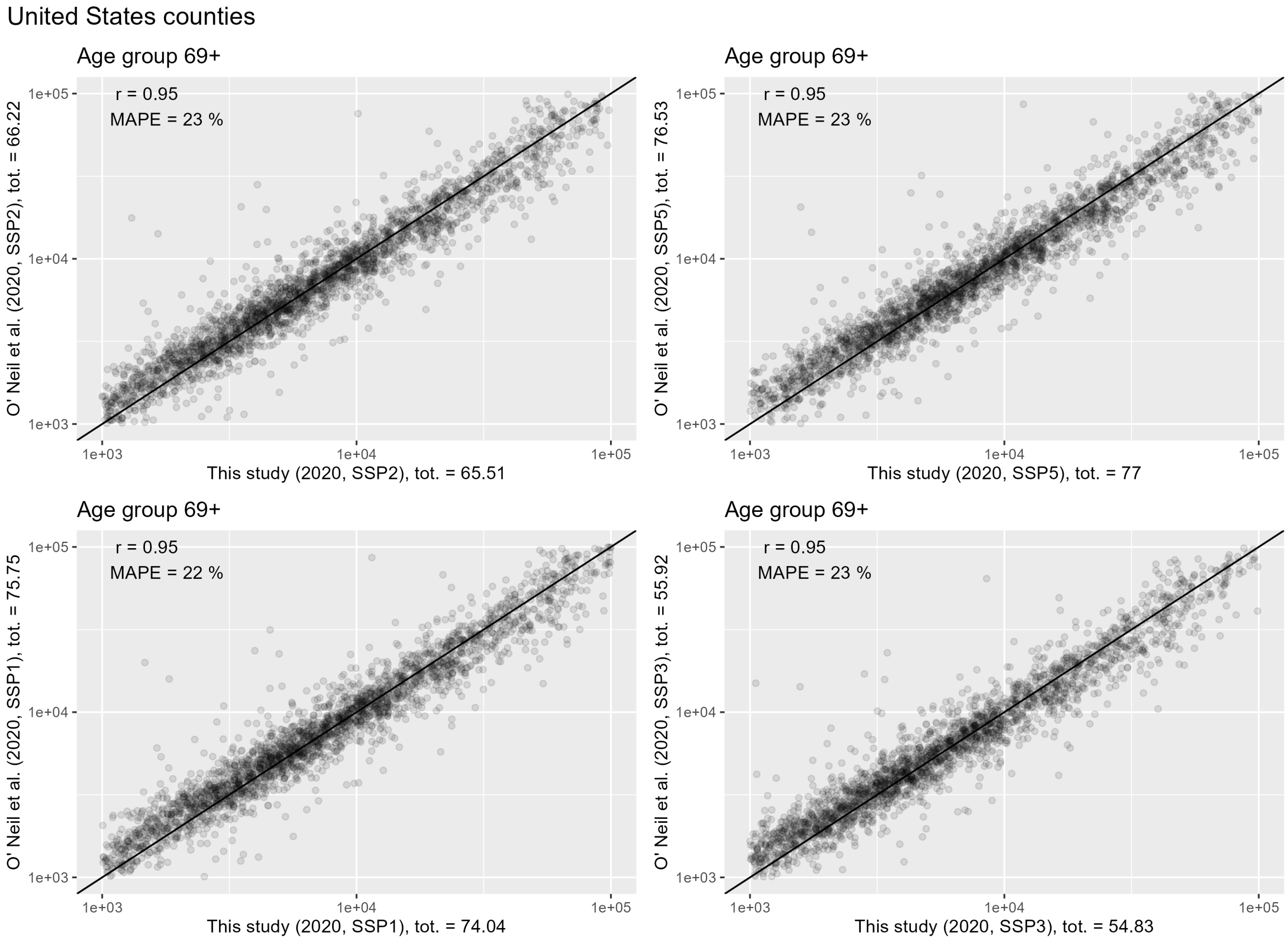

Figure SI-1. Comparison of the age-stratified population projections against values estimated in Striessing et al.[77].

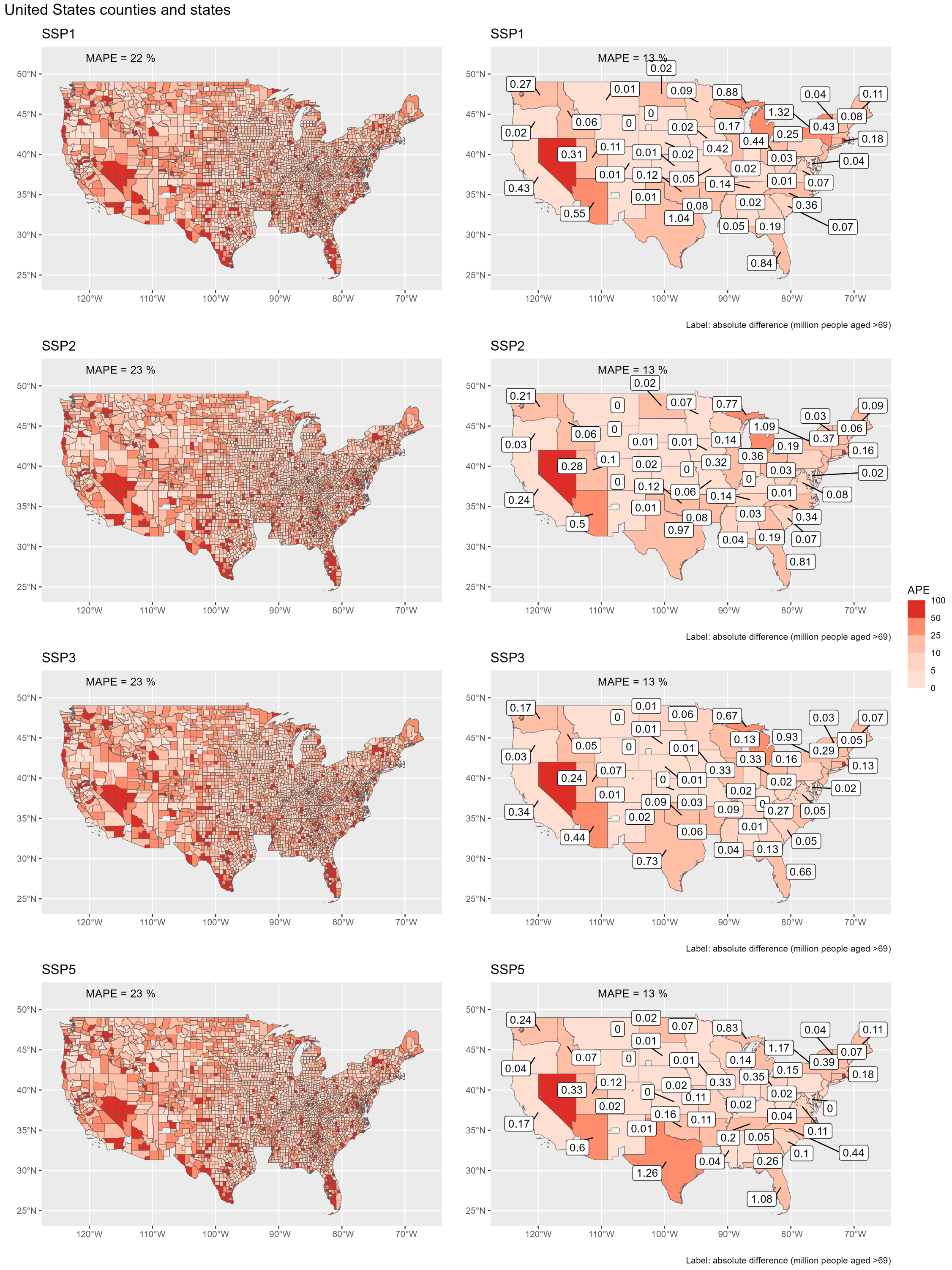

Figure SI-2. Comparison of the age-stratified population projections against values estimated in Striessing et al.[77].

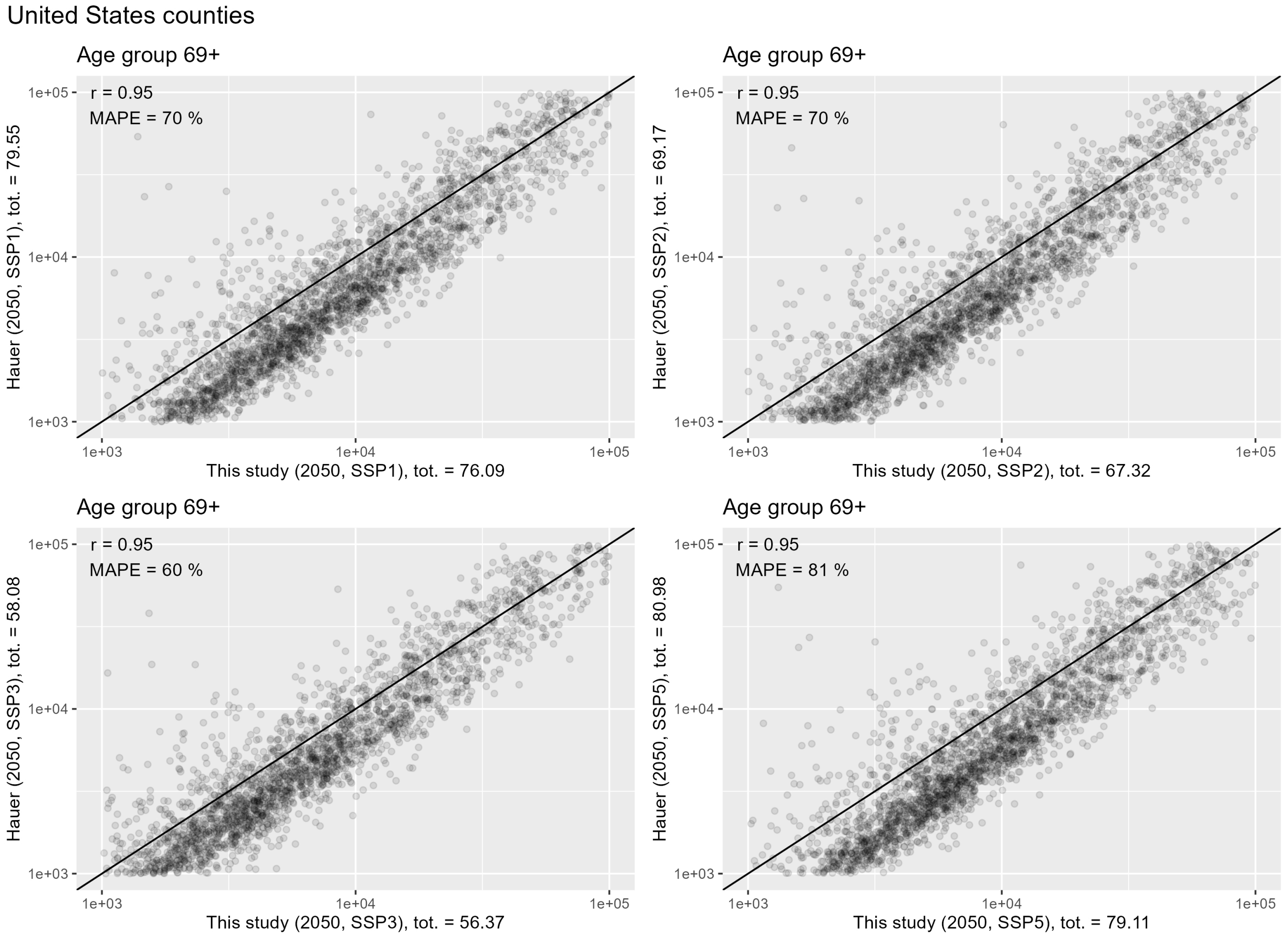

Figure SI-3. Comparison of the age-stratified population projections against values estimated in Hauer[79].

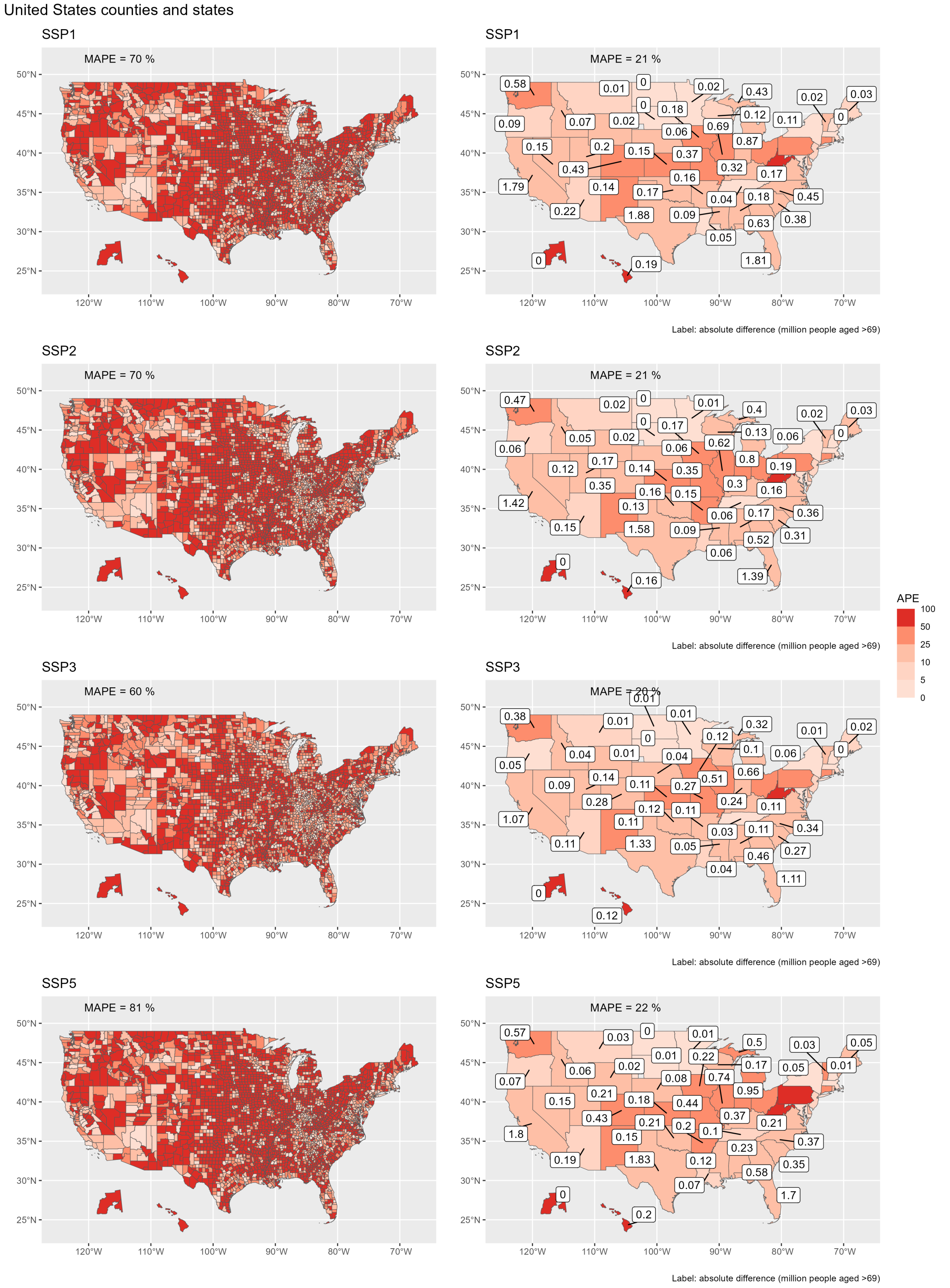

Figure SI-4. Comparison of the age-stratified population projections against values estimated in Hauer[79].

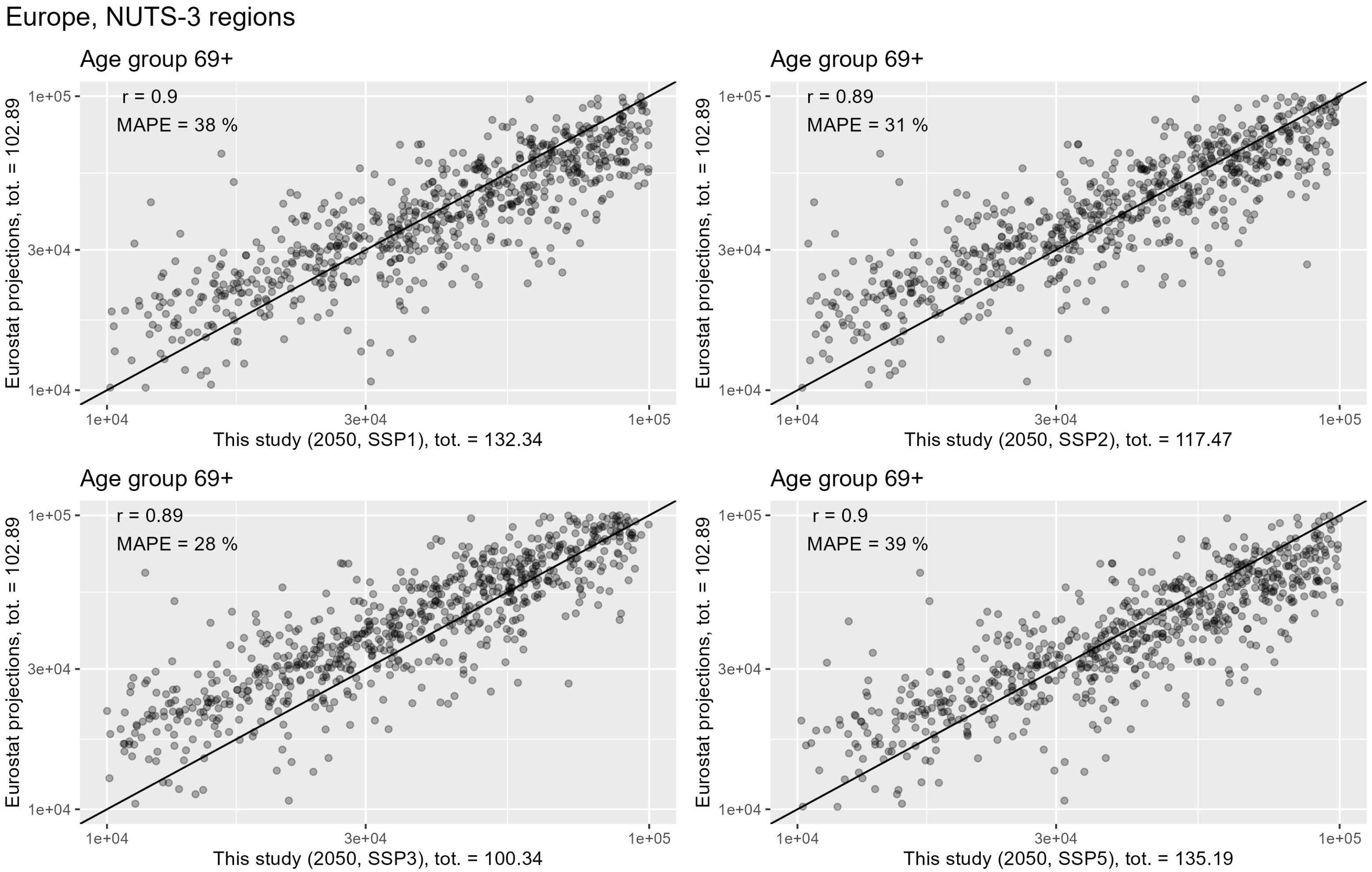

Figure SI-5. Comparison of the age-stratified population projections against values estimated in Eurostat[78].

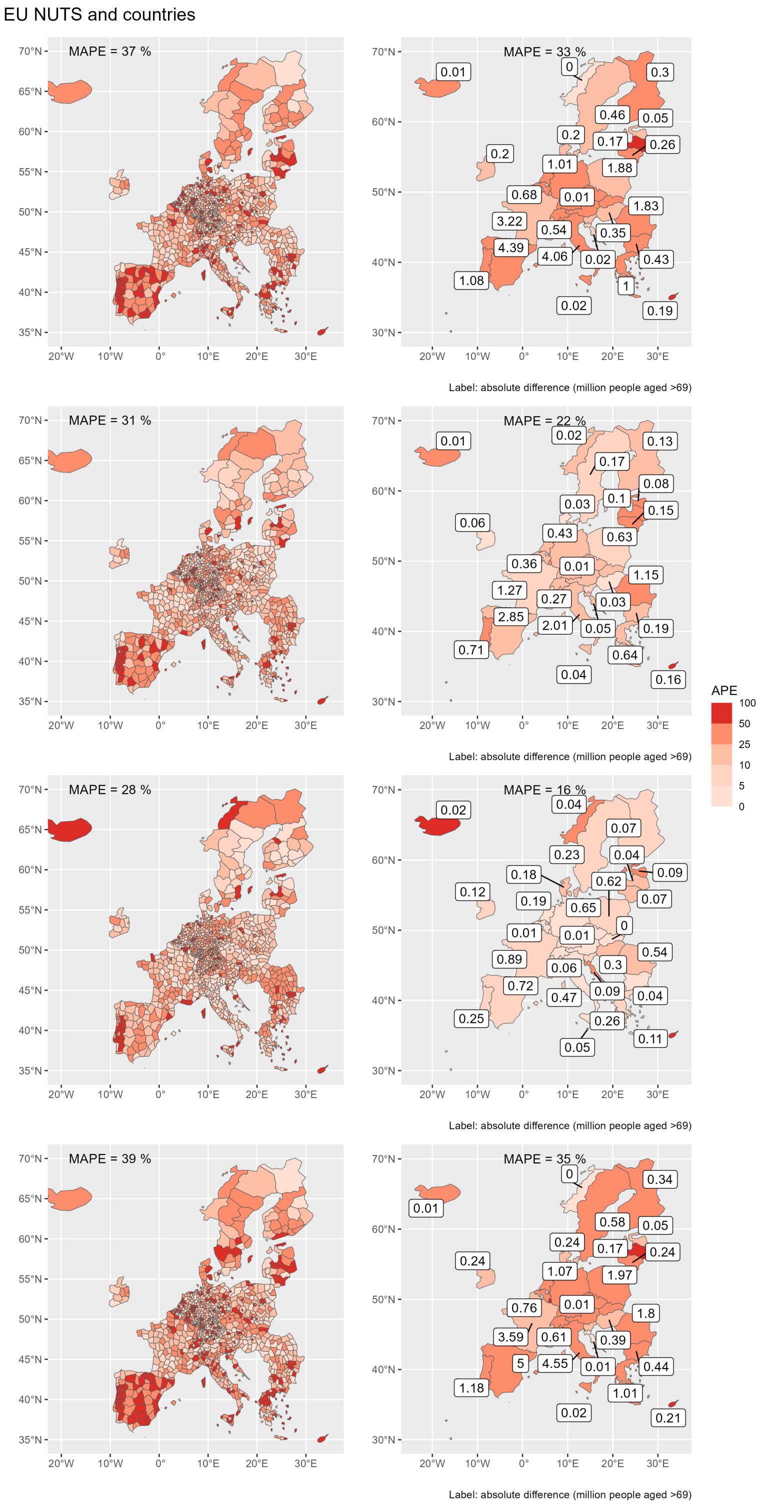

Figure SI-6. Comparison of the age-stratified population projections against values estimated in Eurostat[78].

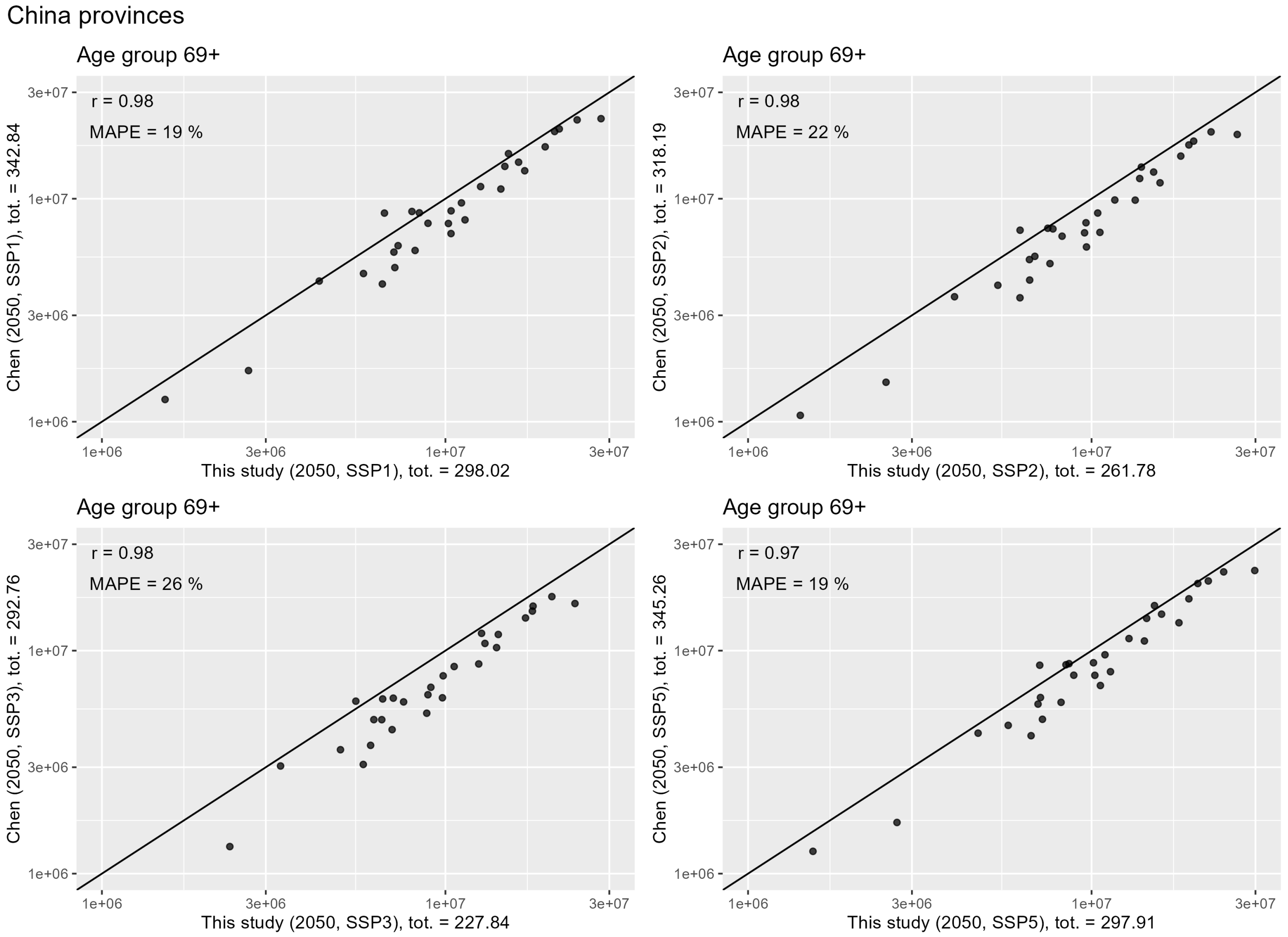

Figure SI-7. Comparison of the age-stratified population projections against values estimated in Chen et al.[82].

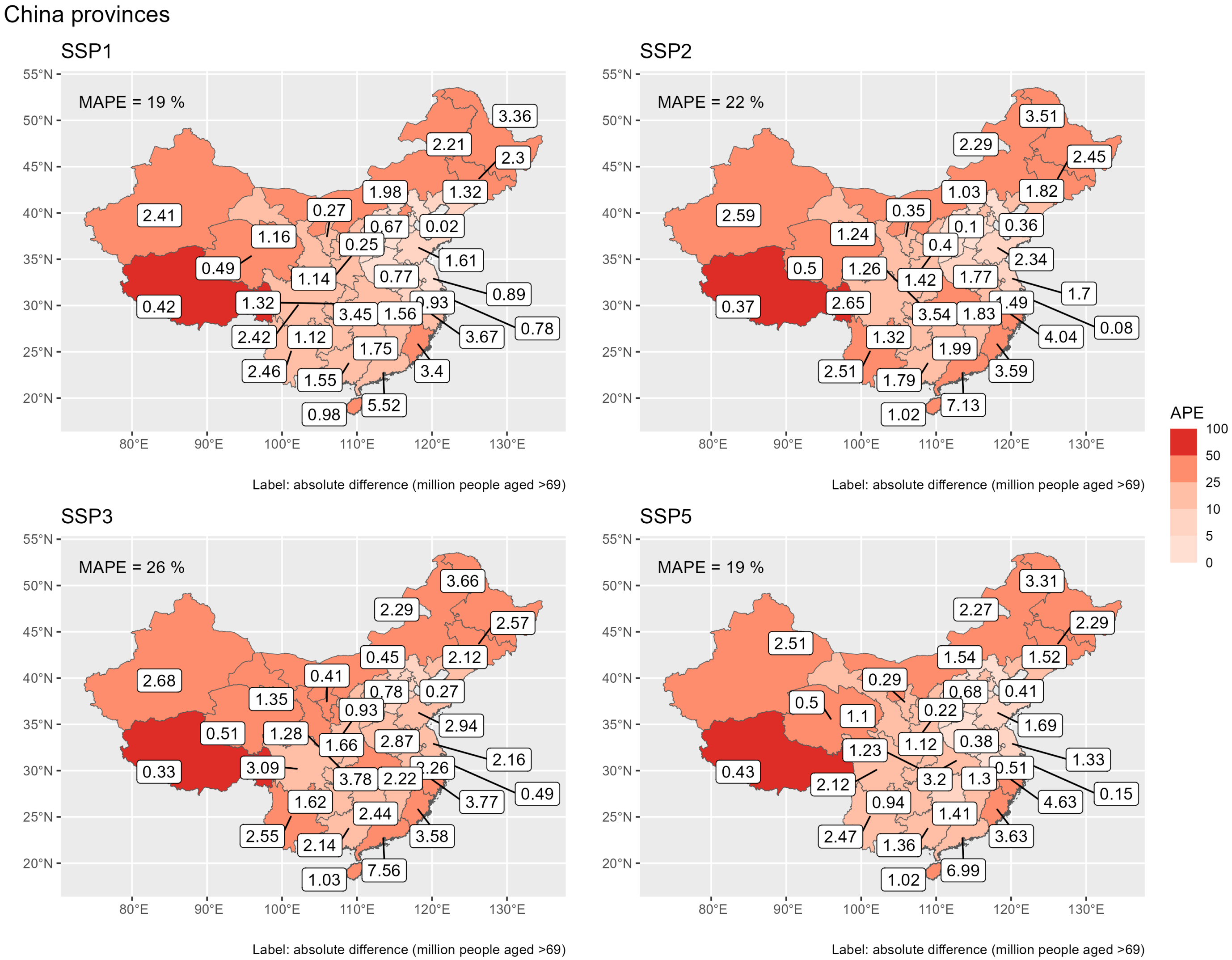

Figure SI-8. Comparison of the age-stratified population projections against values estimated in Chen et al.[82].

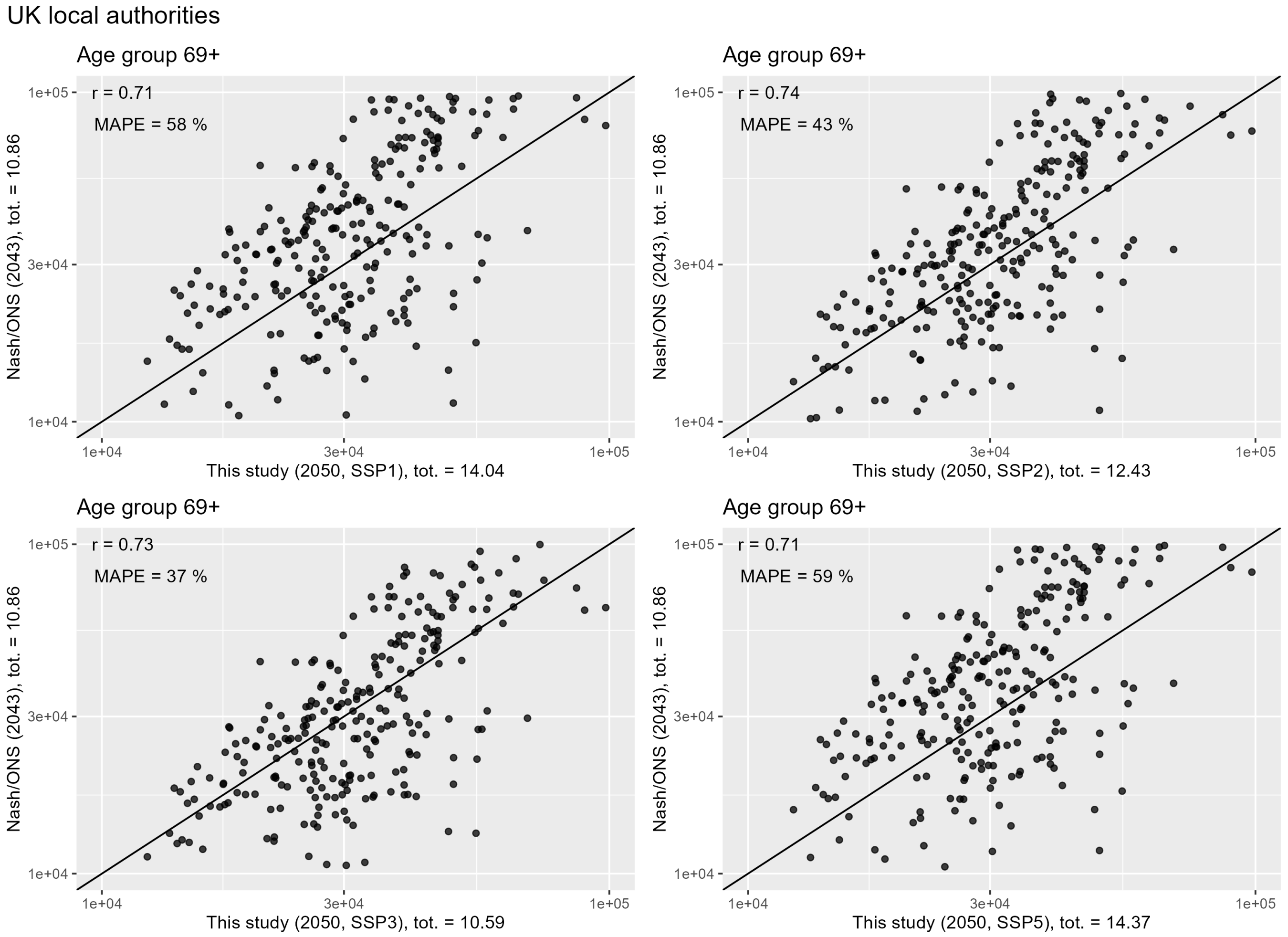

Figure SI-9. Comparison of the age-stratified population projections against values estimated in Nash[81].

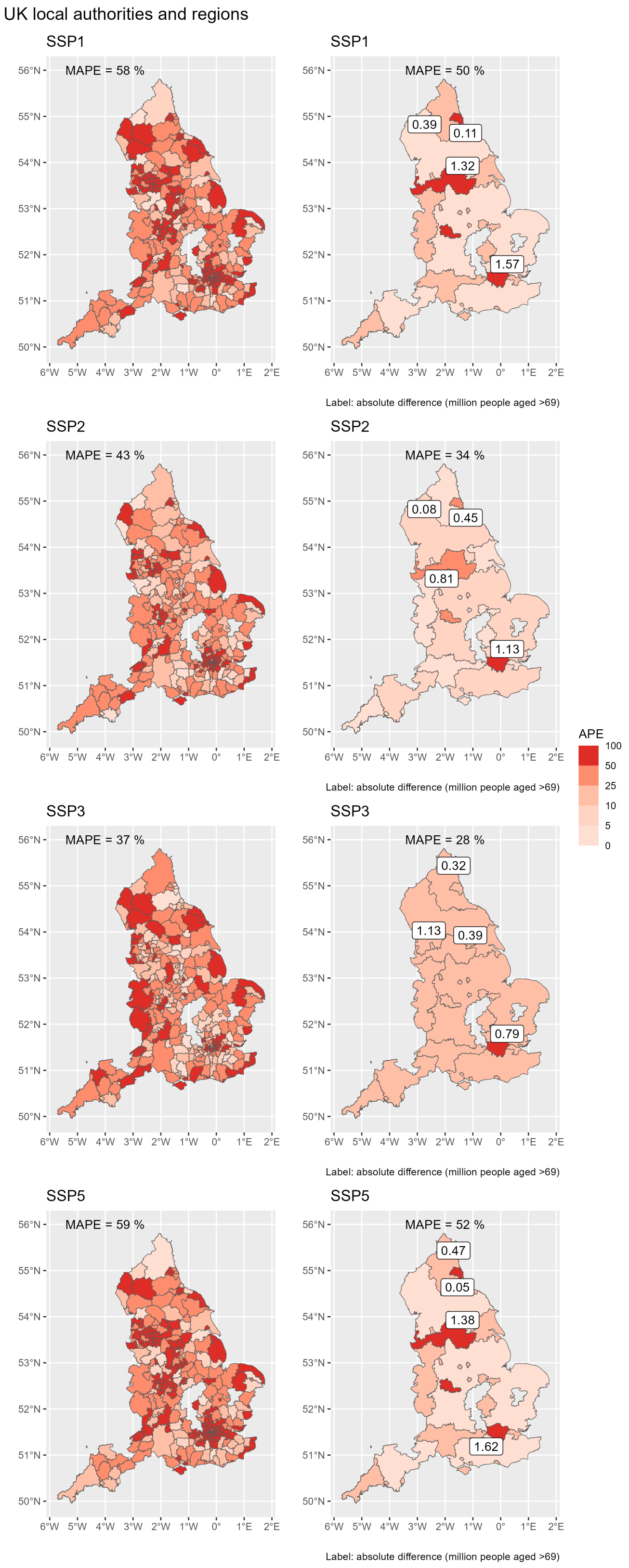

Figure SI-10. Comparison of the age-stratified population projections against values estimated in Nash[81].

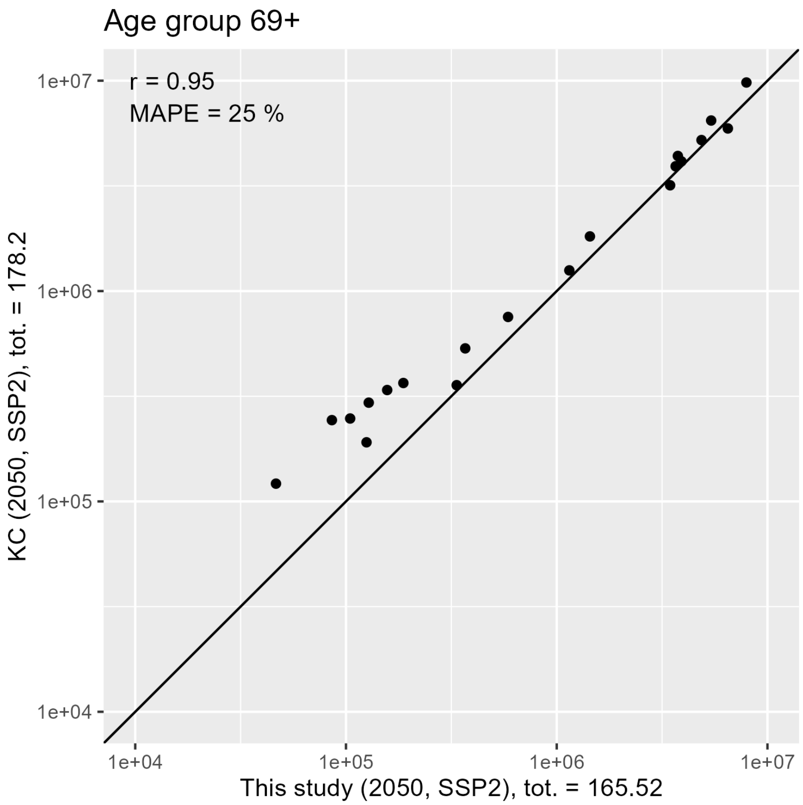

Figure SI-11. Comparison of the age-stratified population projections against values estimated in KC et al.[80].

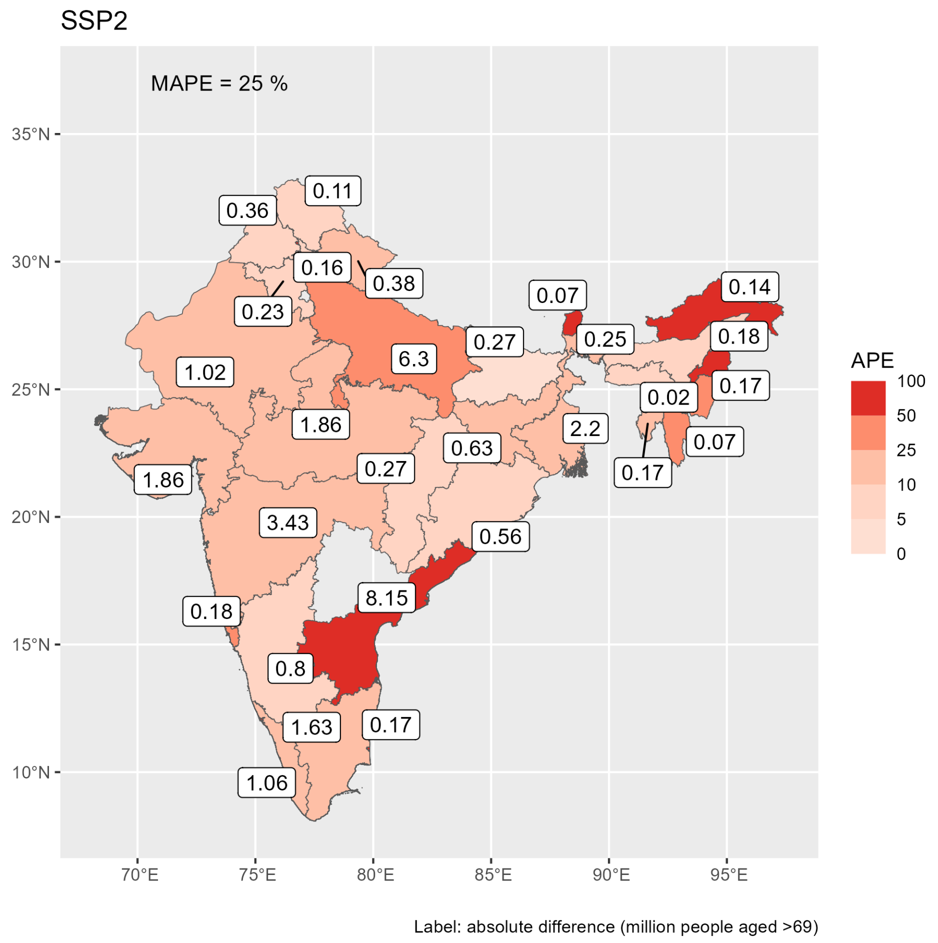

Figure SI-12. Comparison of the age-stratified population projections against values estimated in KC et al.[80].

Supplementary results

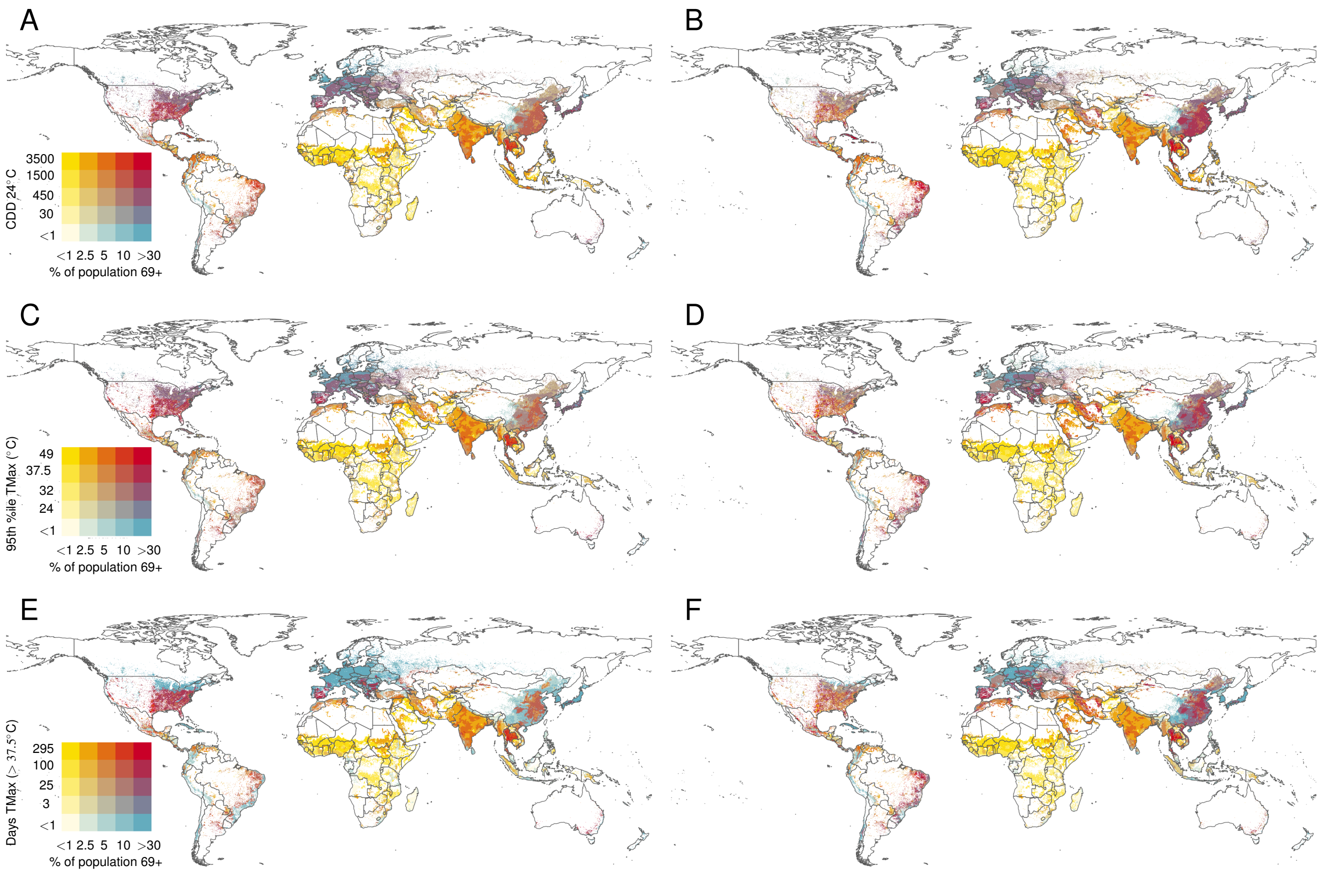

Figure SI-13. Global intersection of aging and heat exposure in the current climate (left column) and circa 2050, SSP5(85) (right column). Proportion of population aged 69+ exposed to annual Cooling Degree Days (A, B), annual temperatures corresponding to the 95th percentile of local extreme heat exposure (C,D), and annual days with $TMax > 37.5°C$ (E,F).

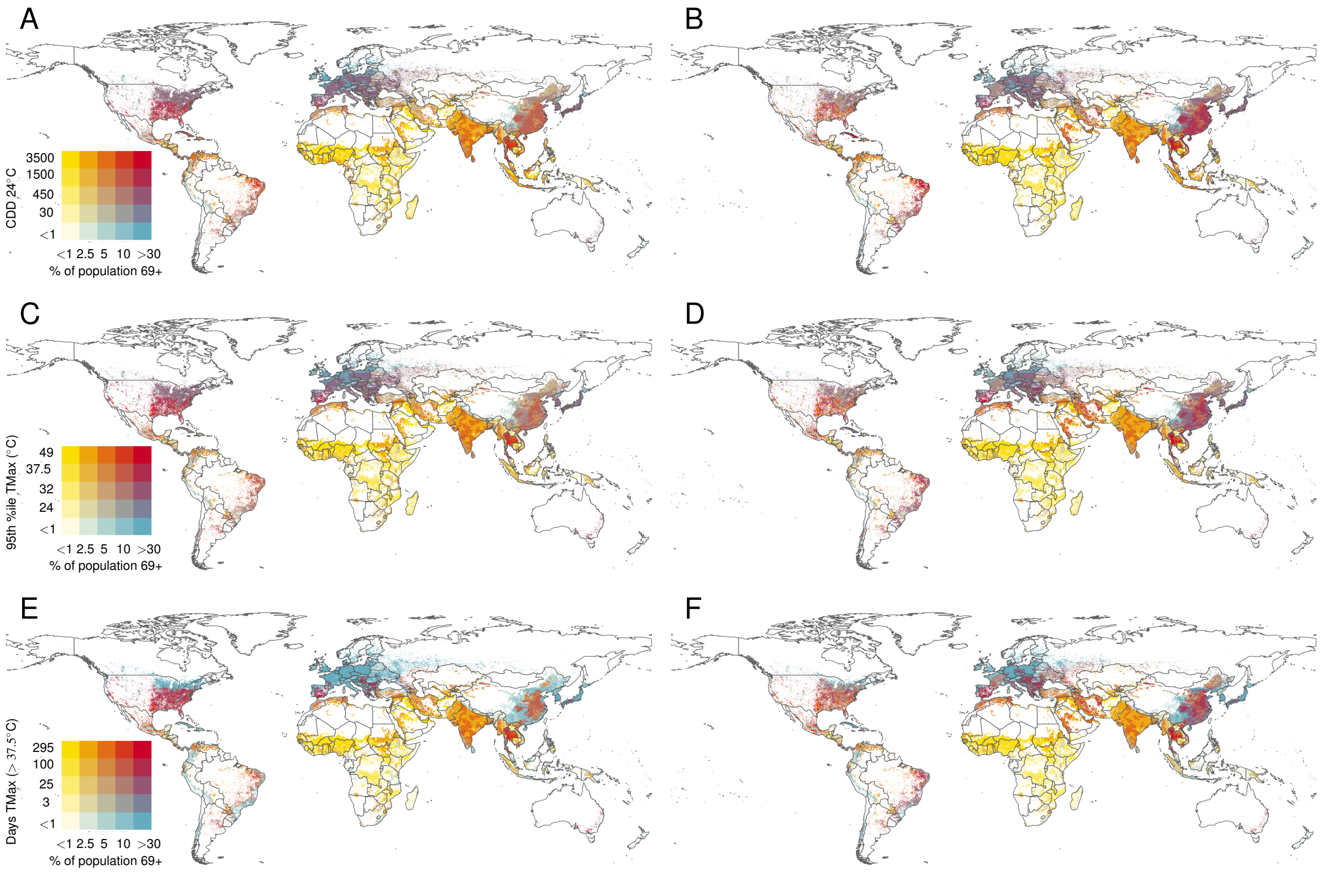

Figure SI-14. Global intersection of aging and heat exposure in the current climate (left column) and circa 2050, SSP1(26) (right column). Proportion of population aged 69+ exposed to annual Cooling Degree Days (A, B), annual temperatures corresponding to the 95th percentile of local extreme heat exposure (C,D), and annual days with $TMax > 37.5°C$ (E,F).

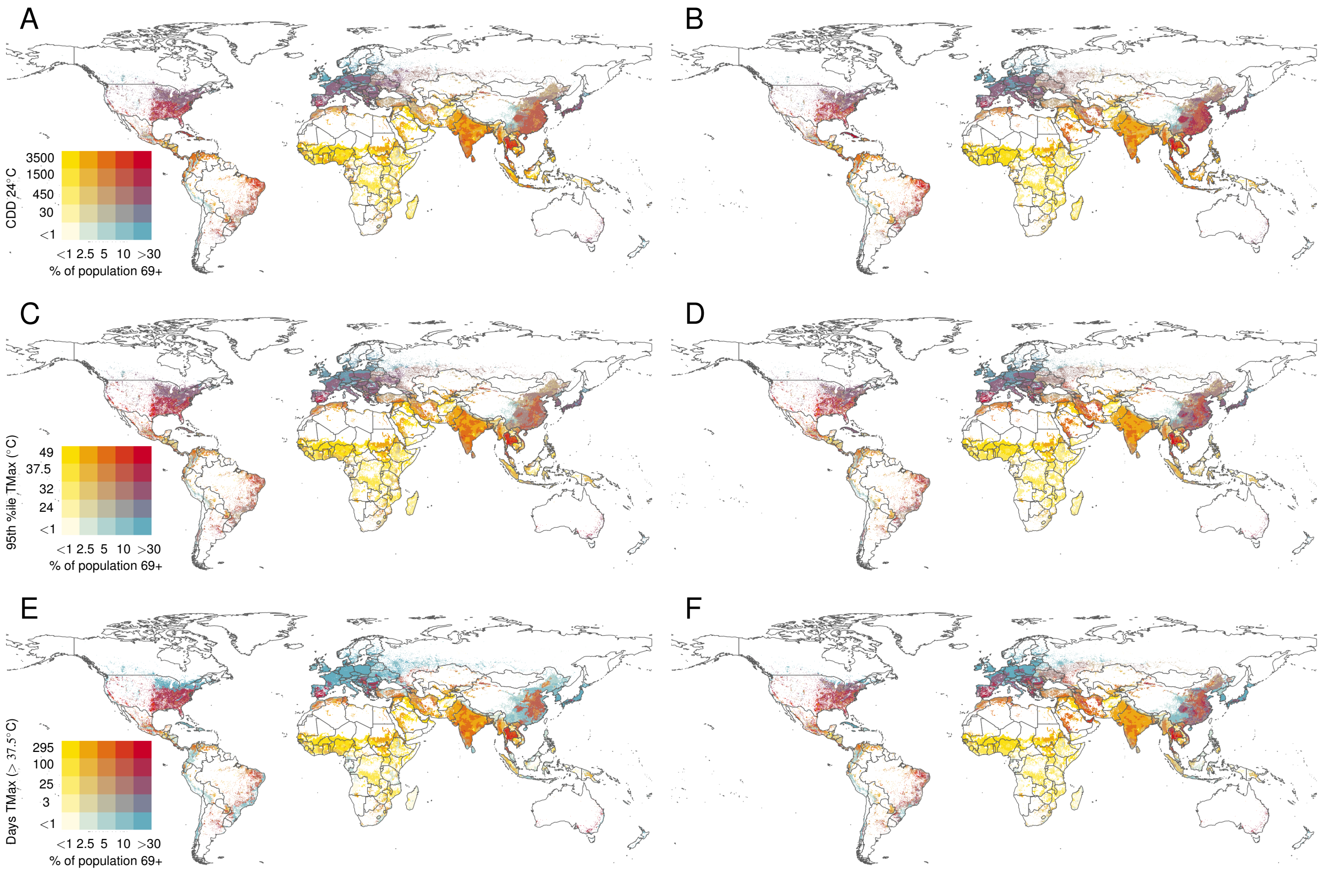

Figure SI-15. Global intersection of aging and heat exposure in the current climate (left column) and circa 2050, SSP3(70) (right column). Proportion of population aged 69+ exposed to annual Cooling Degree Days (A, B), annual temperatures corresponding to the 95th percentile of local extreme heat exposure (C,D), and annual days with $TMax > 37.5°C$ (E,F).

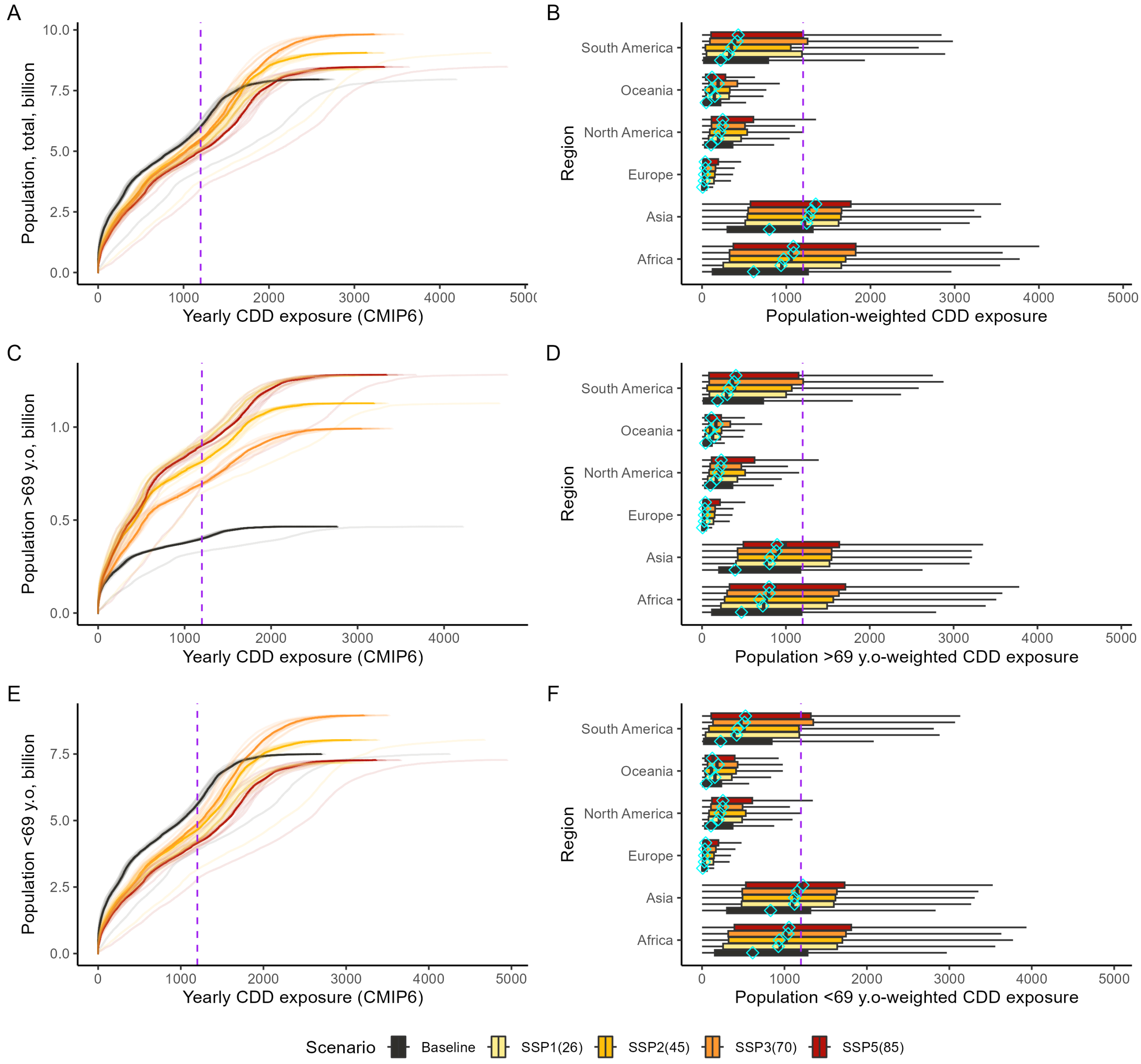

Figure SI-16. (A,C,E) Cumulative count of global population (total; >69 years old., <69 years old) exposed to a given amount of median CDDs/yr. (historical vs. 2050 for SSPs 2(45) and 5(85), CMIP6 GCMs (with 'hot models'[53] excluded) range: light lines, multi-model median: bold lines.). (B,D,F) Boxplots of region-specific population-weighted (total; >69 years old., <69 years old) exposure to a given amount of median CDDs/yr (historical vs. 2050 for SSPs 2(45) and 5(85), CMIP6 GCMs (with 'hot models'[53] excluded) range and multi-model median highlighted with a diamond.).

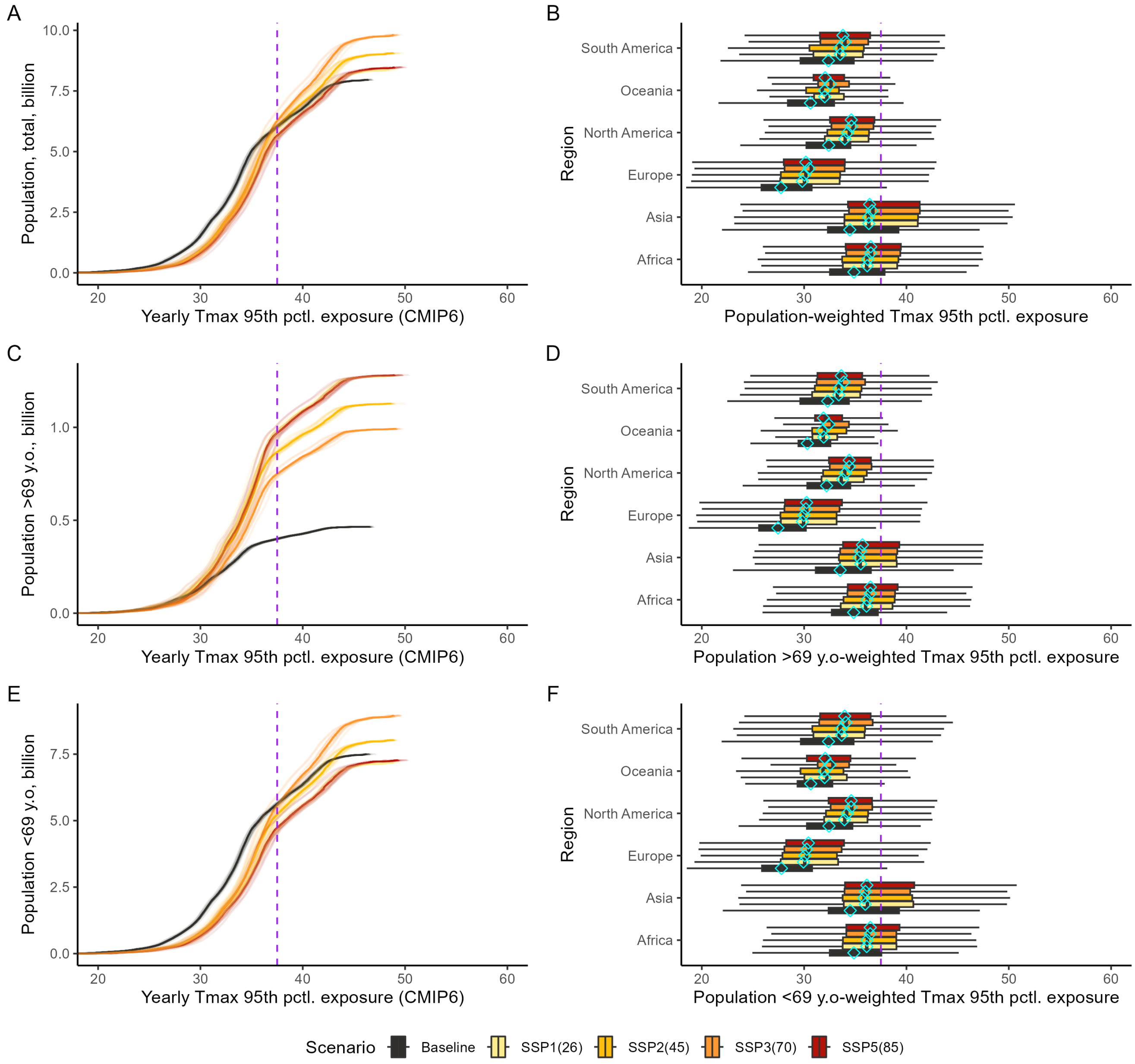

Figure SI-17. (A,C,E) Cumulative count of global population (total; >69 years old., <69 years old) exposed to a given °C corresponding to the 95th percentile of acute extreme heat exposure. (historical vs. 2050 for SSPs 2(45) and 5(85), CMIP6 GCMs (with 'hot models'[53] excluded) range: light lines, multi-model median: bold lines.). (B,D,F) Boxplots of region-specific population-weighted (total; >69 years old., <69 years old) exposure to a given °C corresponding to the 95th percentile of acute extreme heat exposure (historical vs. 2050 for SSPs 2(45) and 5(85), CMIP6 GCMs (with 'hot models'[53] excluded) range and multi-model median highlighted with a diamond.).

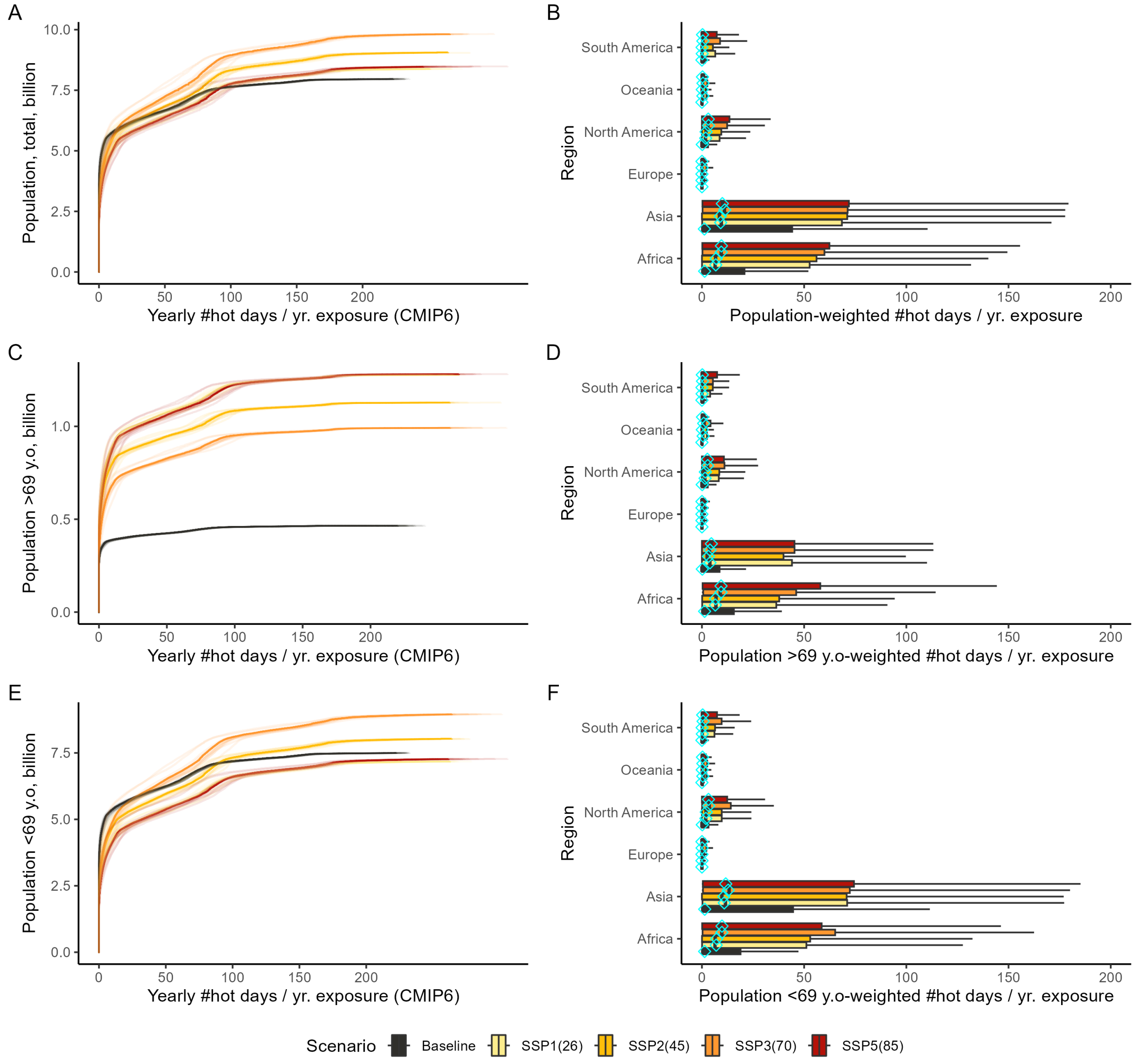

Figure SI-18. (A,C,E) Cumulative count of global population (total; >69 years old., <69 years old) exposed to a given #*hotdays* / yr. (defined as days where $TMAX > 37.5°C$ (historical vs. 2050 for SSPs 2(45) and 5(85), CMIP6 GCMs (with 'hot models'[53] excluded) range: light lines, multi-model median: bold lines.). (B,D,F) Boxplots of region-specific population-weighted (total; >69 years old., <69 years old) exposure to a given #*hotdays* / yr. (defined as days where $TMAX > 37.5°C$ (historical vs. 2050 for SSPs 2(45) and 5(85), CMIP6 GCMs (with 'hot models'[53] excluded) range and multi-model median highlighted with a diamond.).

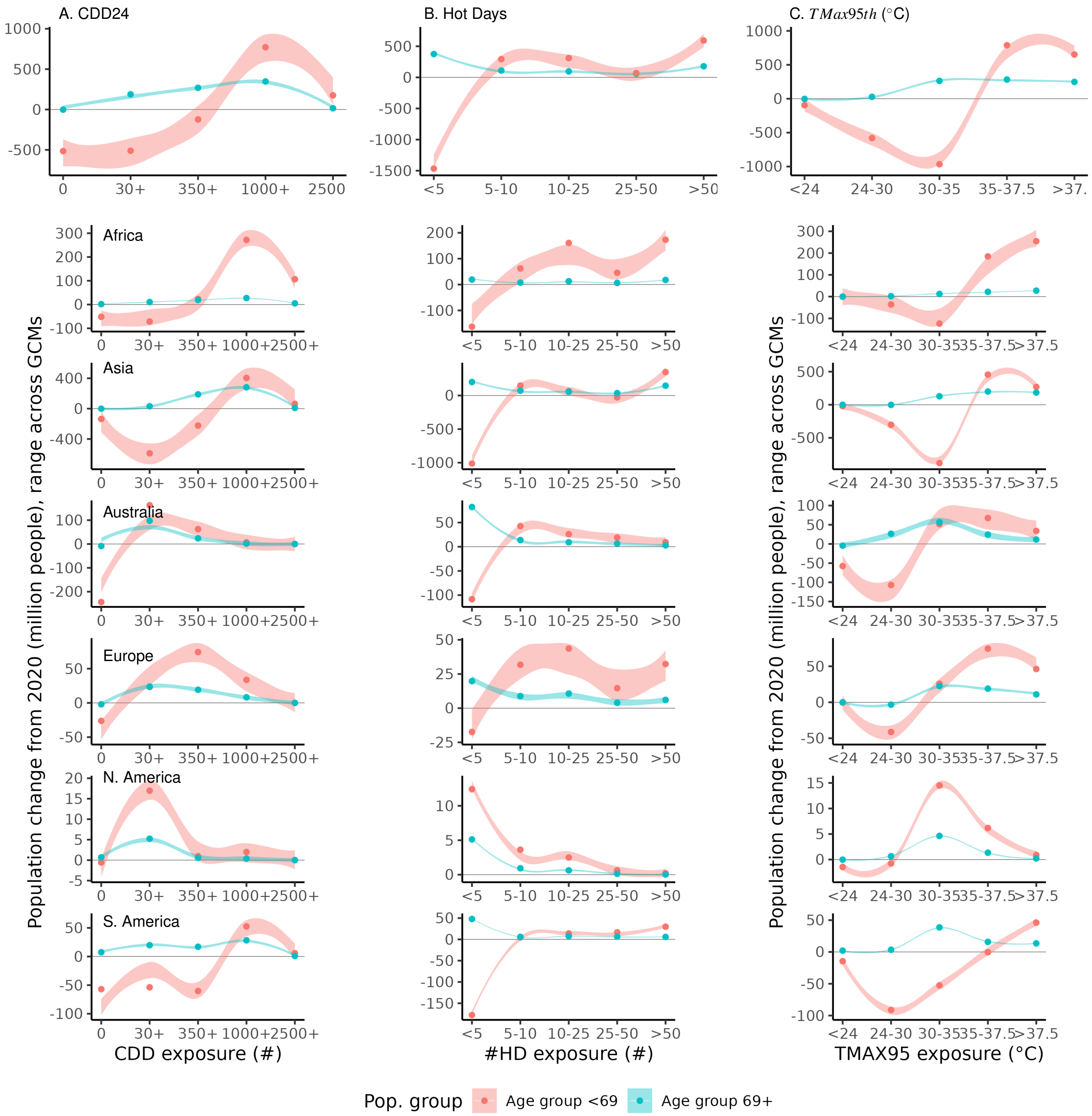

Figure SI-19. Regional and age-group specific trends in the cumulative and intensity of acute exposure of population groups: 2020-2050. (A) GCM uncertainty range for the count of individuals exposed to a given CDD exposure level, age stratification, faceted by region, difference between SSP5(85) and current population. (B) GCM uncertainty range for the count of individuals exposed to a given number of annual days with $TMax > 37.5$°C, age stratification, faceted by region, , difference between SSP5(85) and current population. (C) GCM uncertainty range for the count of individuals exposed to a given 95th percentile maximum temperature exposure level, age stratification, faceted by region, , difference between SSP5(85) and current population.

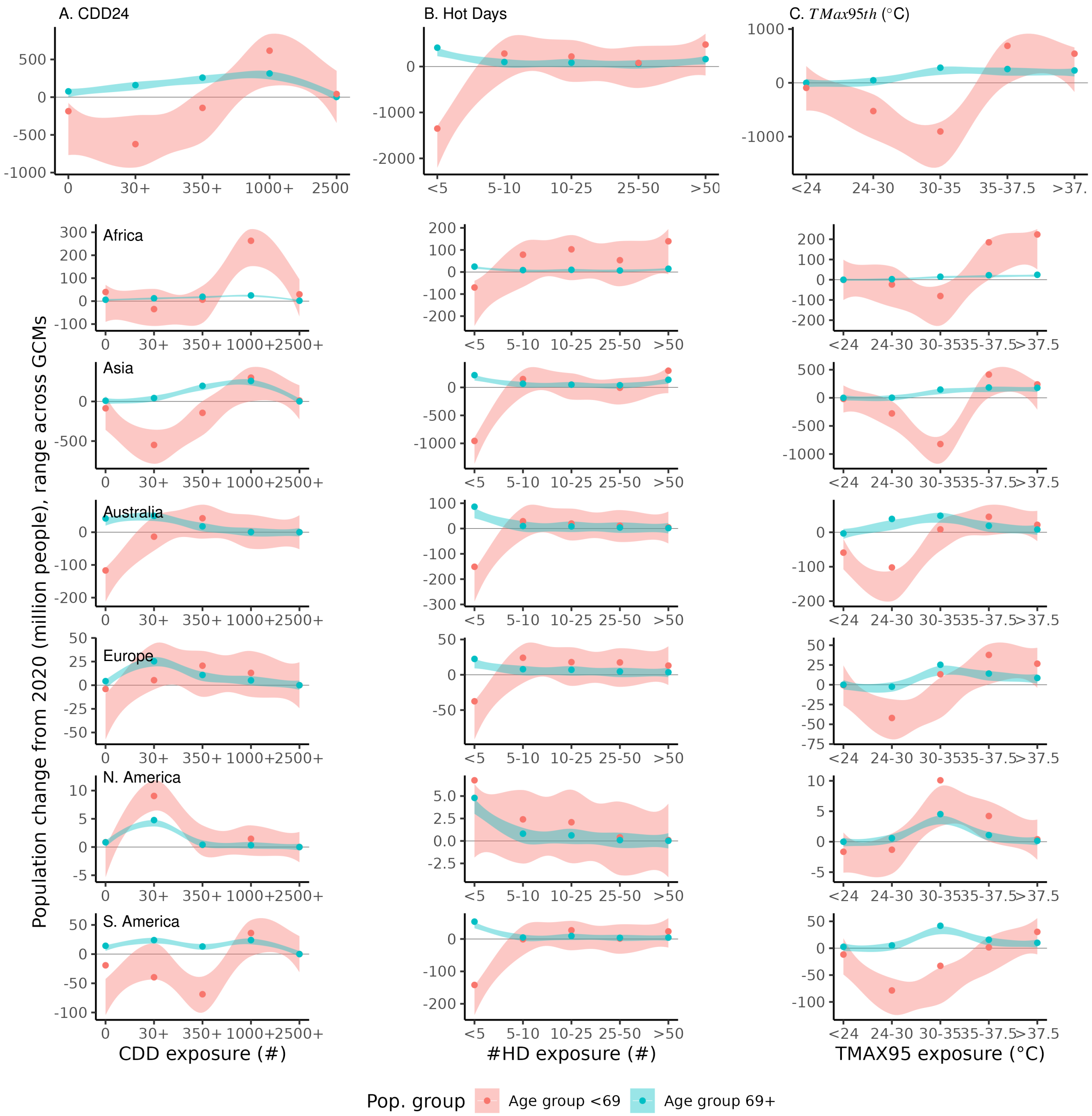

Figure SI-20. Regional and age-group specific trends in the cumulative and intensity of acute exposure of population groups: 2020-2050. (A) GCM uncertainty range for the count of individuals exposed to a given CDD exposure level, age stratification, faceted by region, difference between SSP1(26) and current population. (B) GCM uncertainty range for the count of individuals exposed to a given number of annual days with $TMax > 37.5$°C, age stratification, faceted by region, , difference between SSP1(26) and current population. (C) GCM uncertainty range for the count of individuals exposed to a given 95th percentile maximum temperature exposure level, age stratification, faceted by region, , difference between SSP1(26) and current population.

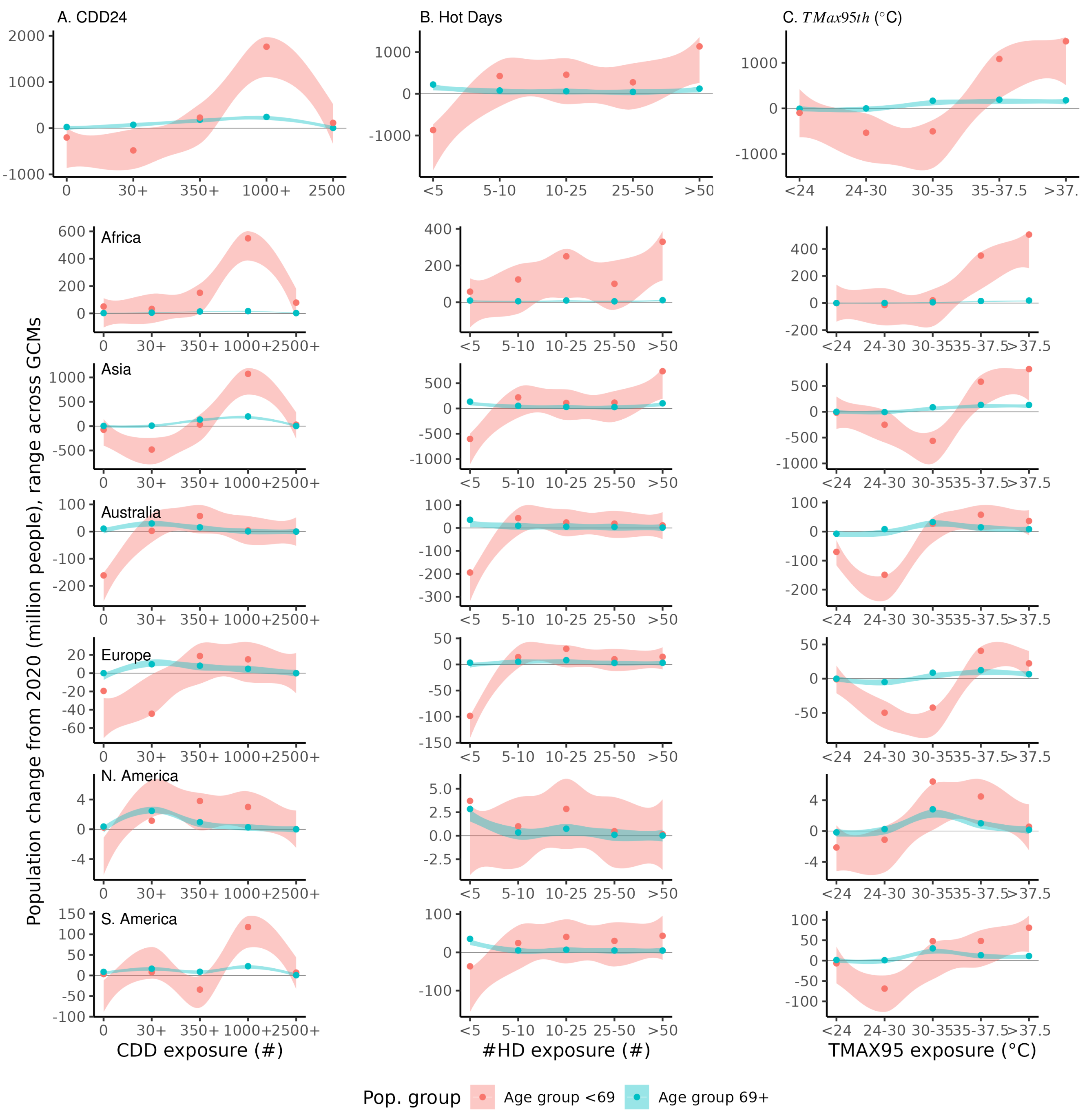

Figure SI-21. Regional and age-group specific trends in the cumulative and intensity of acute exposure of population groups: 2020-2050. (A) GCM uncertainty range for the count of individuals exposed to a given CDD exposure level, age stratification, faceted by region, difference between SSP3(70) and current population. (B) GCM uncertainty range for the count of individuals exposed to a given number of annual days with $TMax > 37.5$°C, age stratification, faceted by region, , difference between SSP3(70) and current population. (C) GCM uncertainty range for the count of individuals exposed to a given 95th percentile maximum temperature exposure level, age stratification, faceted by region, , difference between SSP3(70) and current population.

Table SI-1. Evolution of older heat exposure determinants under scenarios SSP1(26) and 3(70). CDDs, TMAX95th, #HDs, PDDs, PHDs, PD95th report the median and the interquartile range in brackets.

	Population (10^6)	69+ (%)	CDD24 #	PDDs (10^9)	TMAX95 (°C)	PD95s (10^6)	#HDs #	PHDs (10^6)
				2050 SSP126				
Africa	1716	5.4	879	82	36.5	3388	34	3175
			[843, 926]	[78, 86]	[36.2, 36.6]	[3363, 3402]	[33, 37]	[3064, 3426]
Asia	4562	16.4	942	706	36.2	27124	25	18812
			[927, 991]	[694, 742]	[36.1, 36.4]	[27045, 27238]	[24, 26]	[18302, 19776]
Australia	54	17.4	213	2	31.5	298	3	28
			[200, 229]	[2, 2]	[31.3, 31.8]	[296, 300]	[2, 3]	[23, 29]
Europe	884	24.4	113	24	30.4	6563	3	629
			[94, 123]	[20, 27]	[30.2, 30.7]	[6516, 6623]	[2, 3]	[514, 697]
N. America	453	19.3	310	27	34.2	2982	10	885
			[289, 349]	[25, 30]	[33.7, 34.5]	[2944, 3007]	[9, 11]	[815, 938]
S. America	665	17	599	68	32.9	3729	7	809
			[574, 625]	[65, 71]	[32.8, 33.1]	[3719, 3754]	[7, 7]	[766, 840]
World	8387	15.2	709	906	34.8	44459	19	24485
			[693, 755]	[885, 964]	[34.5, 34.9]	[44127, 44636]	[19, 20]	[23700, 25455]
				2050 SSP370				
Africa	2249	3	1007	69	36.9	2525	40	2718
			[941, 1030]	[64, 70]	[36.7, 37]	[2512, 2533]	[38, 40]	[2568, 2769]
Asia	5445	10.8	997	585	36.3	21332	27	15675
			[925, 1018]	[543, 598]	[36.1, 36.4]	[21196, 21396]	[26, 27]	[15050, 16062]
Australia	48	14.8	247	2	32.1	230	4	26
			[237, 278]	[2, 2]	[32, 32.1]	[229, 230]	[4, 4]	[25, 29]
Europe	820	19.8	127	21	30.6	4978	4	564
			[110, 133]	[18, 22]	[30.4, 30.9]	[4949, 5017]	[3, 4]	[503, 636]
N. America	365	17.6	364	24	34.5	2223	12	747
			[344, 394]	[22, 25]	[34.4, 34.8]	[2215, 2244]	[11, 13]	[696, 813]
S. America	839	11.3	684	65	33.4	3184	10	930
			[660, 716]	[63, 68]	[33.2, 33.6]	[3165, 3202]	[8, 10]	[796, 1004]
World	9824	10.1	784	778	35	34688	21	20711
			[727, 792]	[722, 786]	[34.8, 35.1]	[34522, 34880]	[20, 21]	[20035, 21191]

Table SI-2. Population-weighted *t-test* of difference in means between age groups CDD exposure, by region and scenario

reg	scen	t	p	sig
Africa	Baseline (historical climate, 2020 population)	98.274	0	***
Africa	SSP1(26), year 2050	97.715	0	***
Africa	SSP2(45), year 2050	104.126	0	***
Africa	SSP3(70), year 2050	101.367	0	***
Africa	SSP5(85), year 2050	107.801	0	***
Asia	Baseline (historical climate, 2020 population)	354.046	0	***
Asia	SSP1(26), year 2050	219.084	0	***
Asia	SSP2(45), year 2050	232.120	0	***
Asia	SSP3(70), year 2050	230.401	0	***
Asia	SSP5(85), year 2050	225.625	0	***
Europe	Baseline (historical climate, 2020 population)	186.998	0	***
Europe	SSP1(26), year 2050	21.440	0	***
Europe	SSP2(45), year 2050	36.636	0	***
Europe	SSP3(70), year 2050	56.576	0	***
Europe	SSP5(85), year 2050	14.103	0	***
North America	Baseline (historical climate, 2020 population)	10.860	0	***
North America	SSP1(26), year 2050	5.313	0	***
North America	SSP2(45), year 2050	10.606	0	***
North America	SSP3(70), year 2050	7.182	0	***
North America	SSP5(85), year 2050	9.813	0	***
Oceania	Baseline (historical climate, 2020 population)	202.256	0	***
Oceania	SSP1(26), year 2050	31.704	0	***
Oceania	SSP2(45), year 2050	36.072	0	***
Oceania	SSP3(70), year 2050	43.002	0	***
Oceania	SSP5(85), year 2050	27.993	0	***
South America	Baseline (historical climate, 2020 population)	61.790	0	***
South America	SSP1(26), year 2050	15.074	0	***
South America	SSP2(45), year 2050	19.231	0	***
South America	SSP3(70), year 2050	17.489	0	***
South America	SSP5(85), year 2050	18.444	0	***

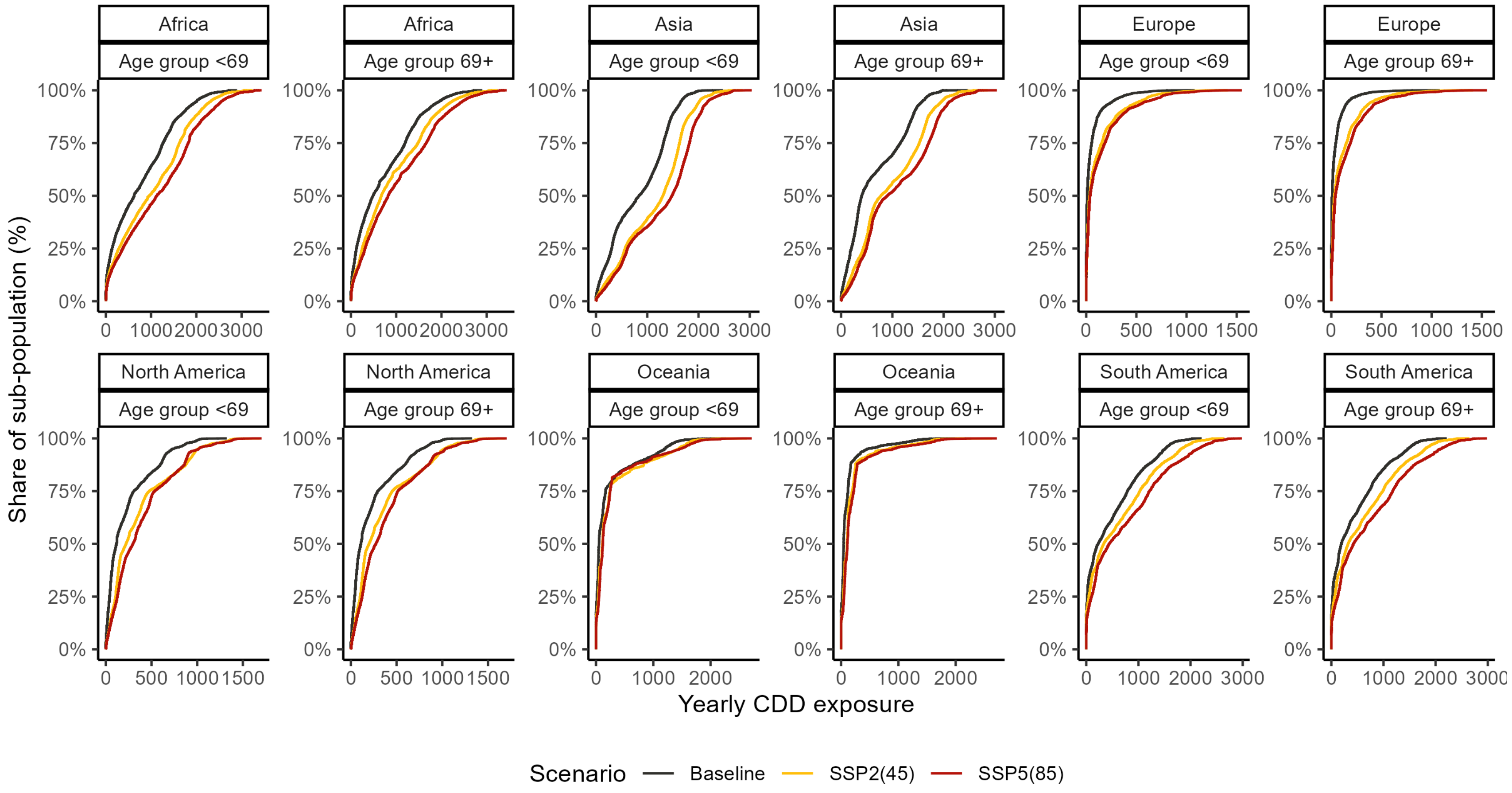

Figure SI-22. Cumulative share of regional population exposed to a given amount of CDDs, age stratification, historical climate.

Table SI-3. Age-group and region-stratified count of people (in millions) by CDDs exposure level, by scenario.

			Age group <69					Age group 69+				
			0-30	30-350	350-1000	1000-2500	>2500	0-30	30-350	350-1000	1000-2500	>2500
	Region	Scenario	Mean	Mean	Mean	Mean	Mean	Mean	Mean	Mean	Mean	Mean
value	Africa	Baseline (historical climate, 2020 population)	182	327	343	456	10	4	8	7	9	0
		SSP1(26), year 2050	169	314	340	755	44	9	21	27	34	2
		SSP2(45), year 2050	166	345	427	860	69	6	18	22	28	2
		SSP3(70), year 2050	177	371	491	1035	106	5	14	21	26	3
		SSP5(85), year 2050	113	273	356	758	97	6	19	26	36	4
	Asia	Baseline (historical climate, 2020 population)	255	1078	1056	1891	0	16	93	57	72	0
		SSP1(26), year 2050	107	455	954	2285	12	22	130	260	336	2
		SSP2(45), year 2050	110	508	1058	2606	4	18	118	229	300	0
		SSP3(70), year 2050	120	513	1128	3066	31	16	95	198	275	3
		SSP5(85), year 2050	74	428	896	2372	29	16	122	252	355	4
	Europe	Baseline (historical climate, 2020 population)	509	222	23	0		75	29	2	0	
		SSP1(26), year 2050	348	248	70	2		111	84	20	1	
		SSP2(45), year 2050	349	281	62	3		97	81	12	1	
		SSP3(70), year 2050	300	267	85	5		79	65	18	1	
		SSP5(85), year 2050	333	324	81	5		95	102	22	1	
	North America	Baseline (historical climate, 2020 population)	71	185	72	2		9	23	9	0	
		SSP1(26), year 2050	39	208	99	19		10	51	21	6	
		SSP2(45), year 2050	36	195	119	15		8	43	23	4	
		SSP3(70), year 2050	24	160	96	22		6	35	18	6	
		SSP5(85), year 2050	31	227	151	26		7	49	28	7	
	Oceania	Baseline (historical climate, 2020 population)	11	16	3	3		1	2	0	0	
		SSP1(26), year 2050	8	28	4	4	0	2	7	1	0	0
		SSP2(45), year 2050	9	28	5	4	0	2	6	1	0	0
		SSP3(70), year 2050	8	21	7	6	0	1	5	1	0	0
		SSP5(85), year 2050	9	34	4	4	0	2	7	1	0	0
	South America	Baseline (historical climate, 2020 population)	193	167	175	108		11	11	10	5	
		SSP1(26), year 2050	112	169	114	157	0	22	37	24	30	0
		SSP2(45), year 2050	123	190	142	172	0	19	34	24	26	0
		SSP3(70), year 2050	134	216	141	246	8	16	30	19	29	1
		SSP5(85), year 2050	92	154	113	166	5	19	34	25	33	1

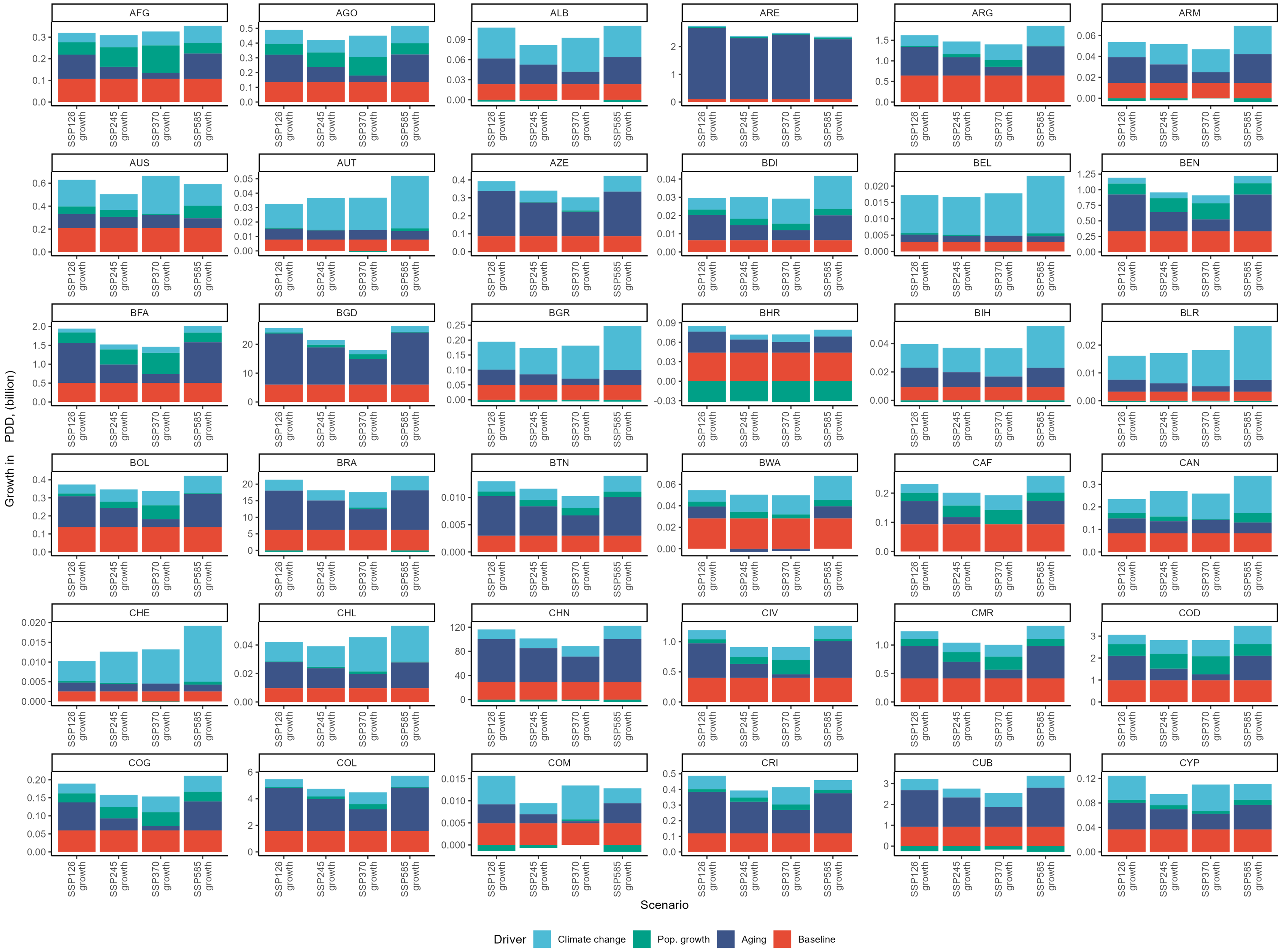

Figure SI-23. Country-level decomposition of determinants of exposure E_r projections, by region and scenario.

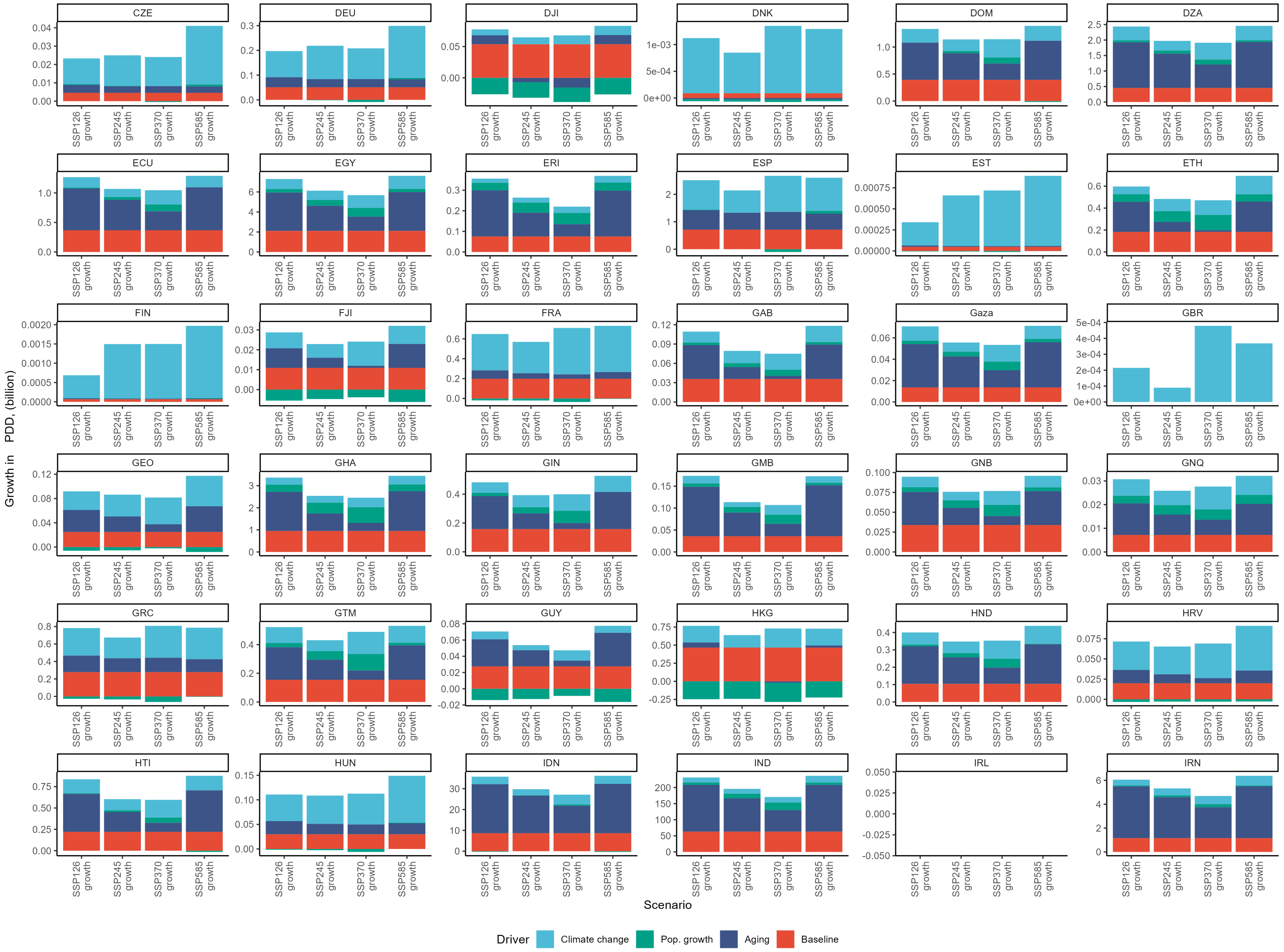

Figure SI-24. Country-level decomposition of determinants of exposure E_r projections, by region and scenario.

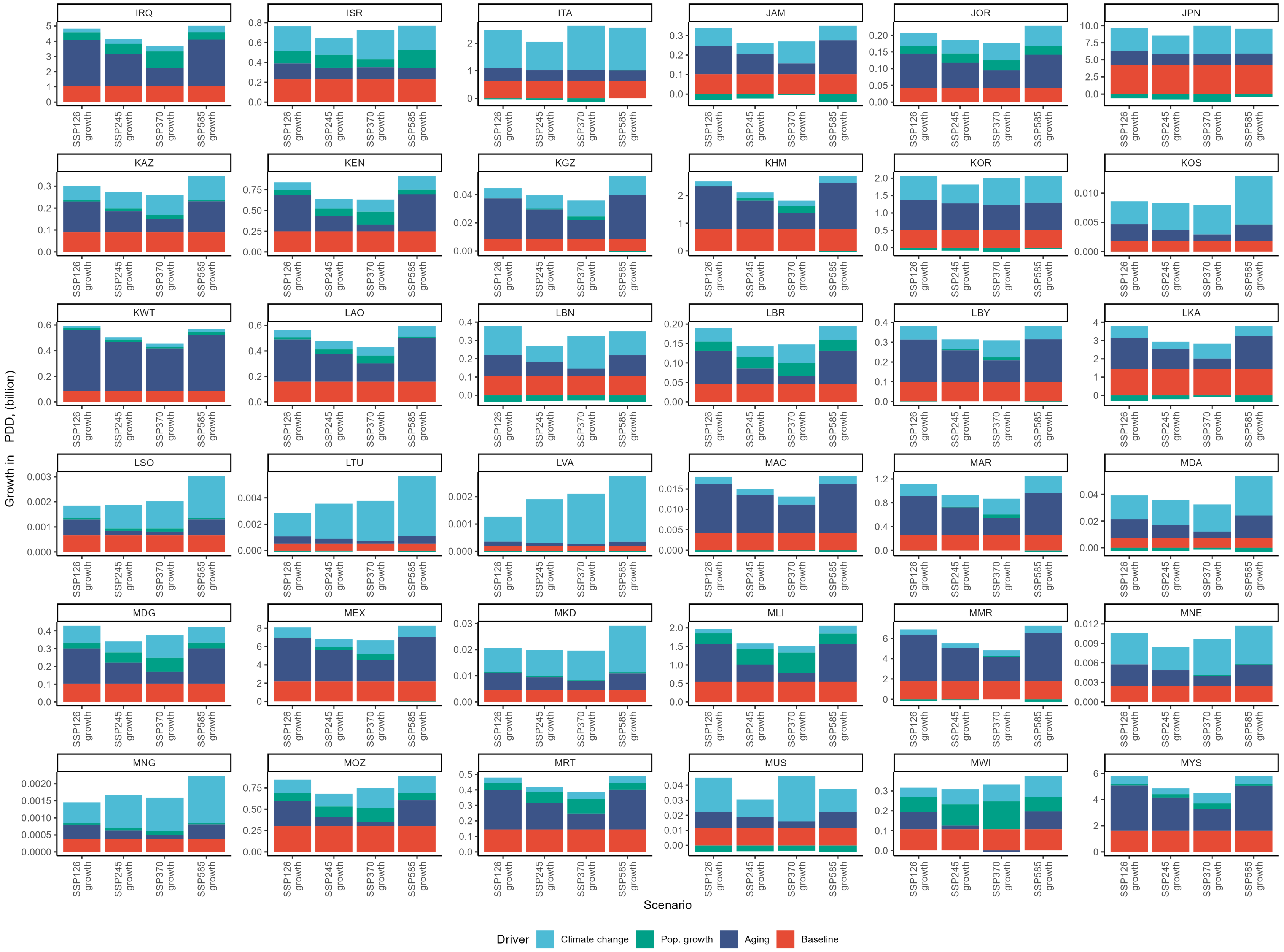

Figure SI-25. Country-level decomposition of determinants of exposure E_r projections, by region and scenario.

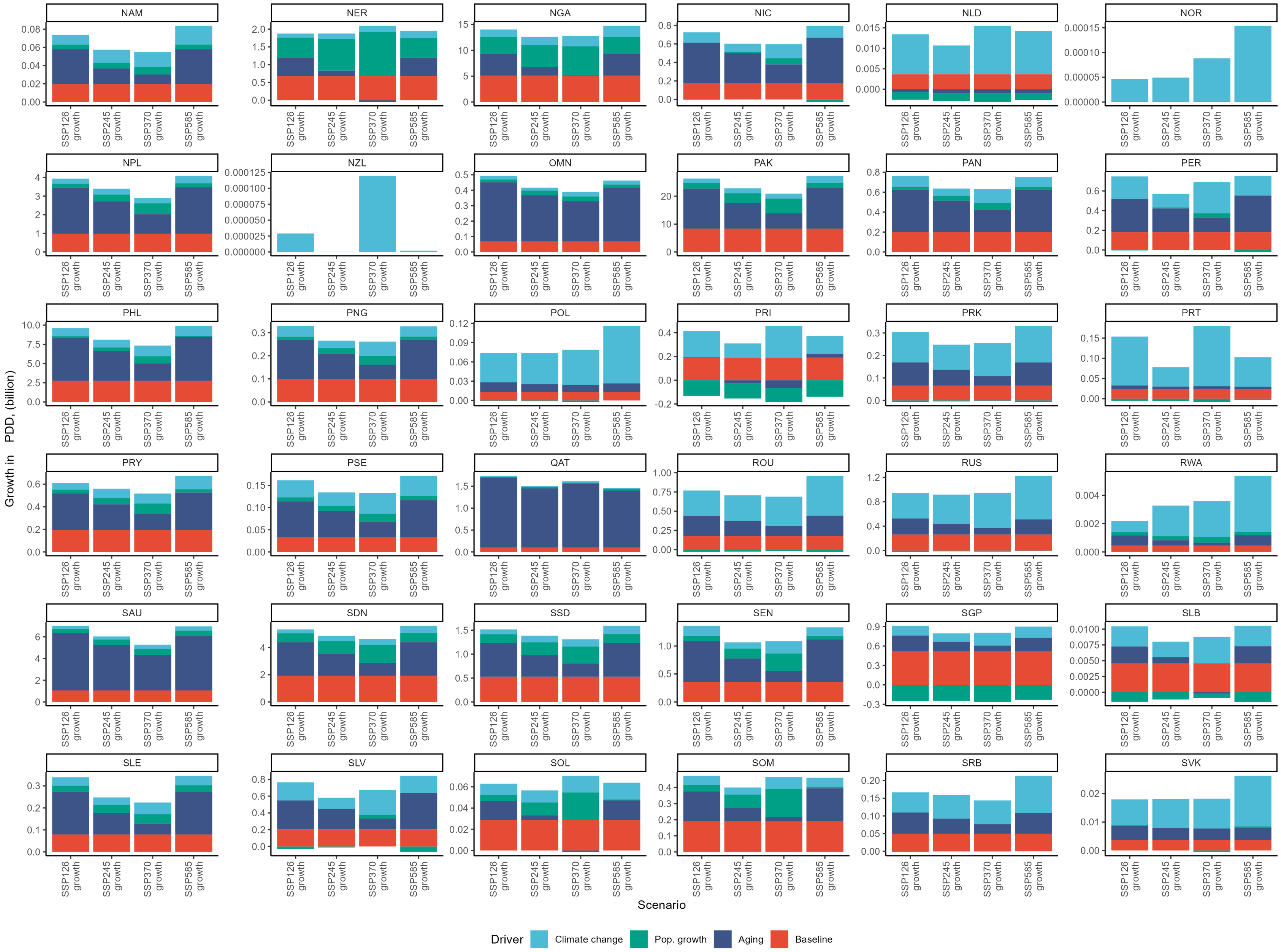

Figure SI-26. Country-level decomposition of determinants of exposure E_r projections, by region and scenario.

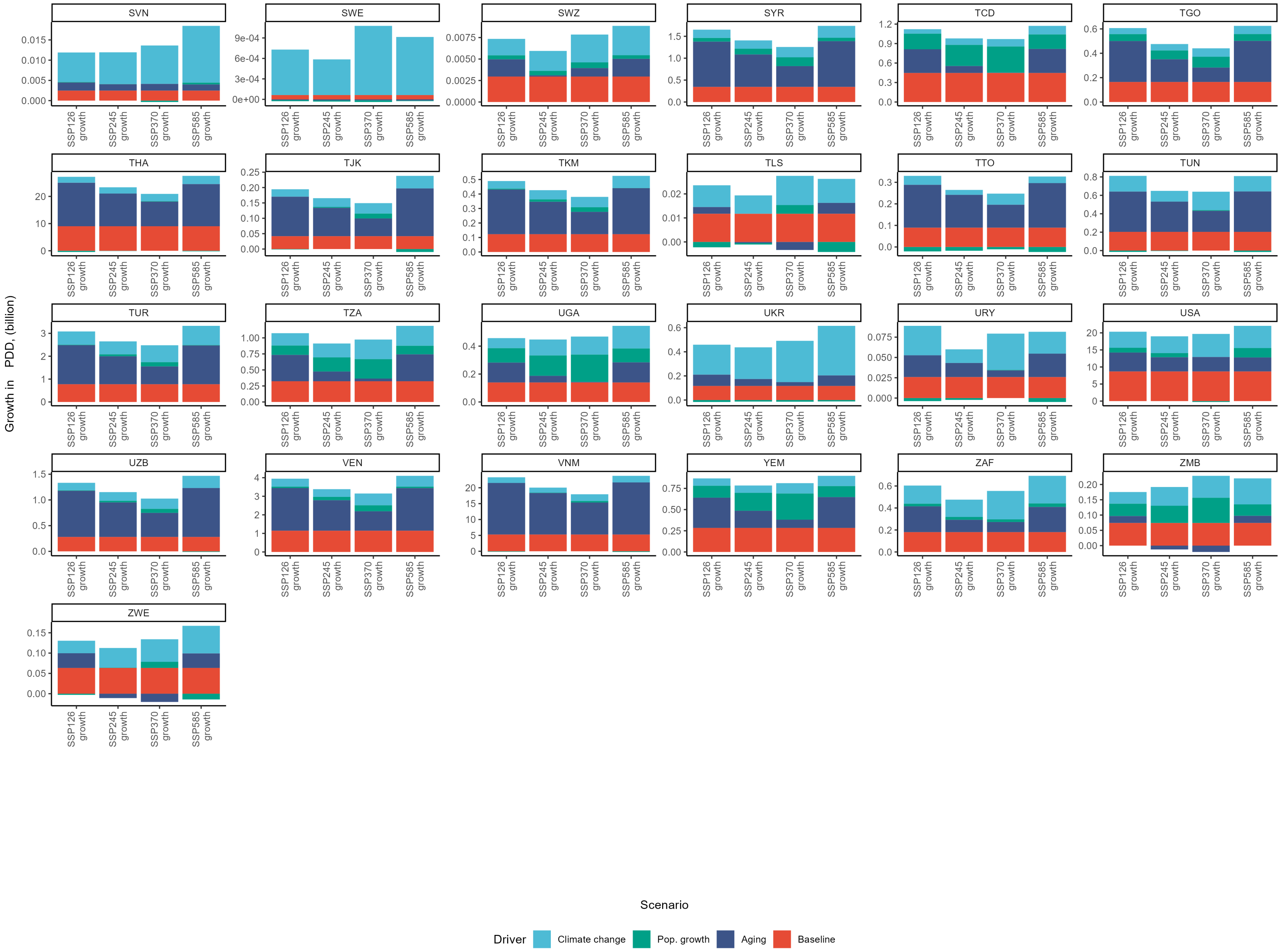

Figure SI-27. Country-level decomposition of determinants of exposure E_r projections, by region and scenario.

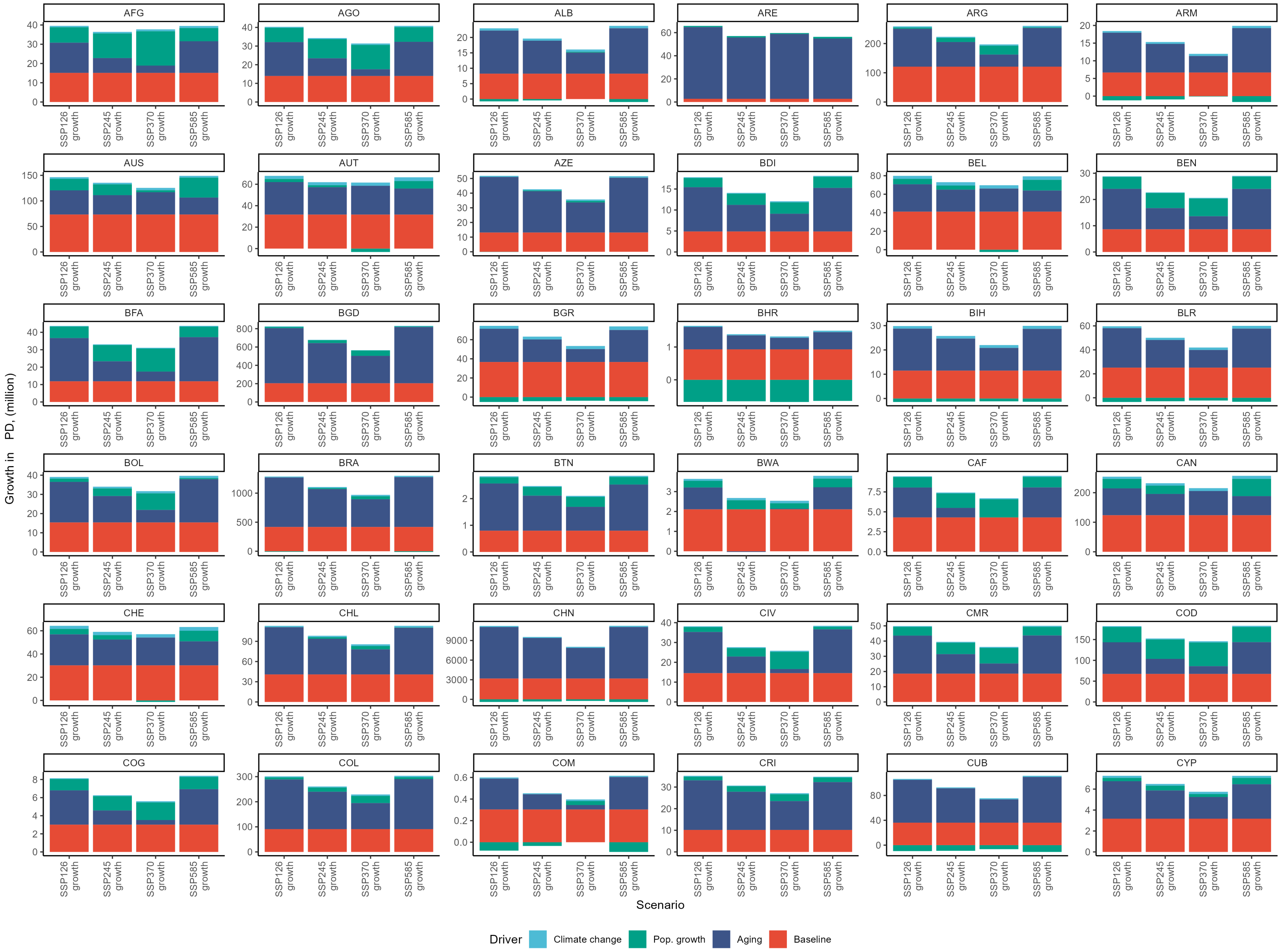

Figure SI-28. Country-level decomposition of determinants of $TMAX_{95}^{th}$ exposure E_r projections, by region and scenario.

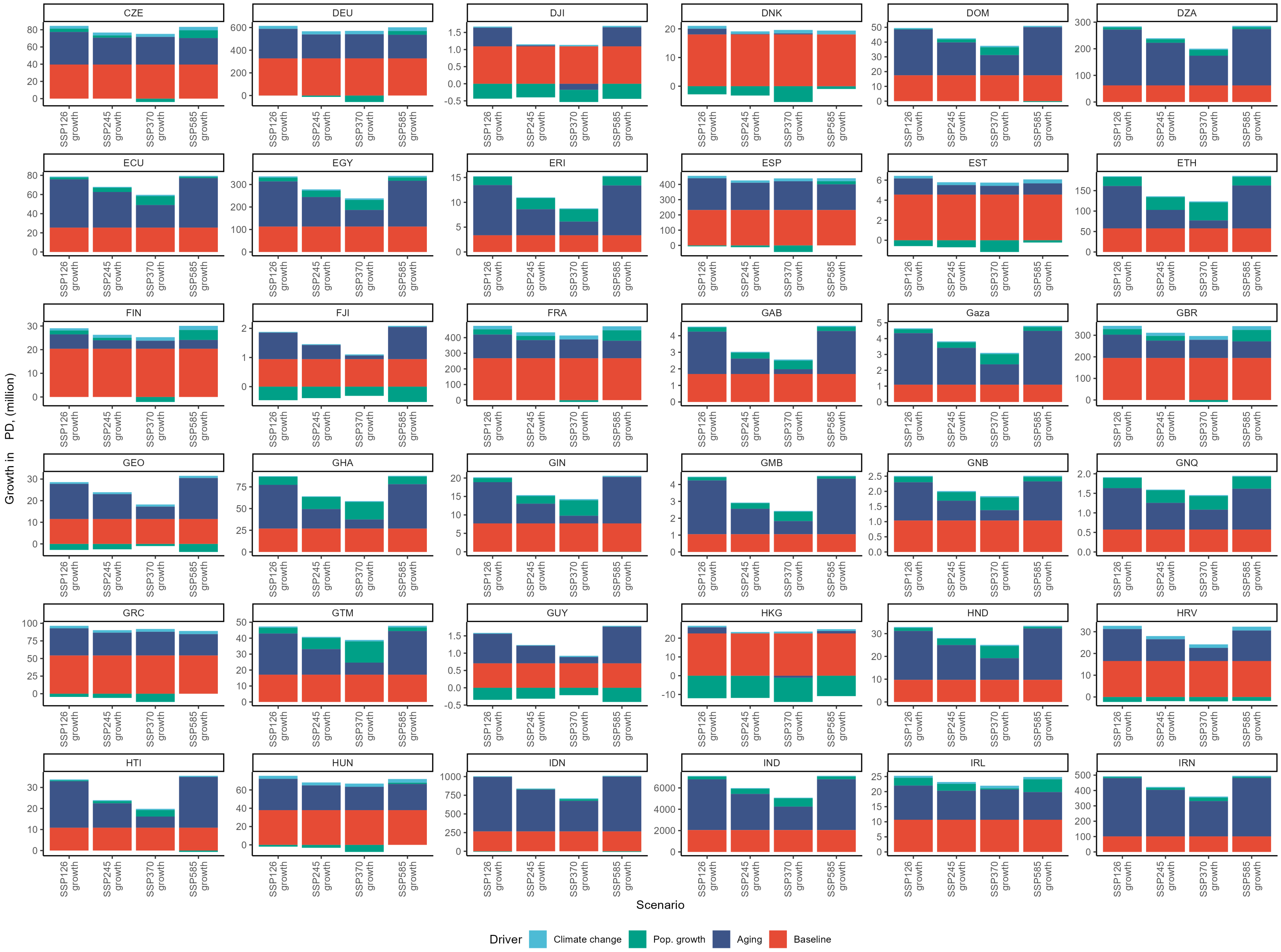

Figure SI-29. Country-level decomposition of determinants of $TMAX_{95}^{th}$ exposure E_r projections, by region and scenario.

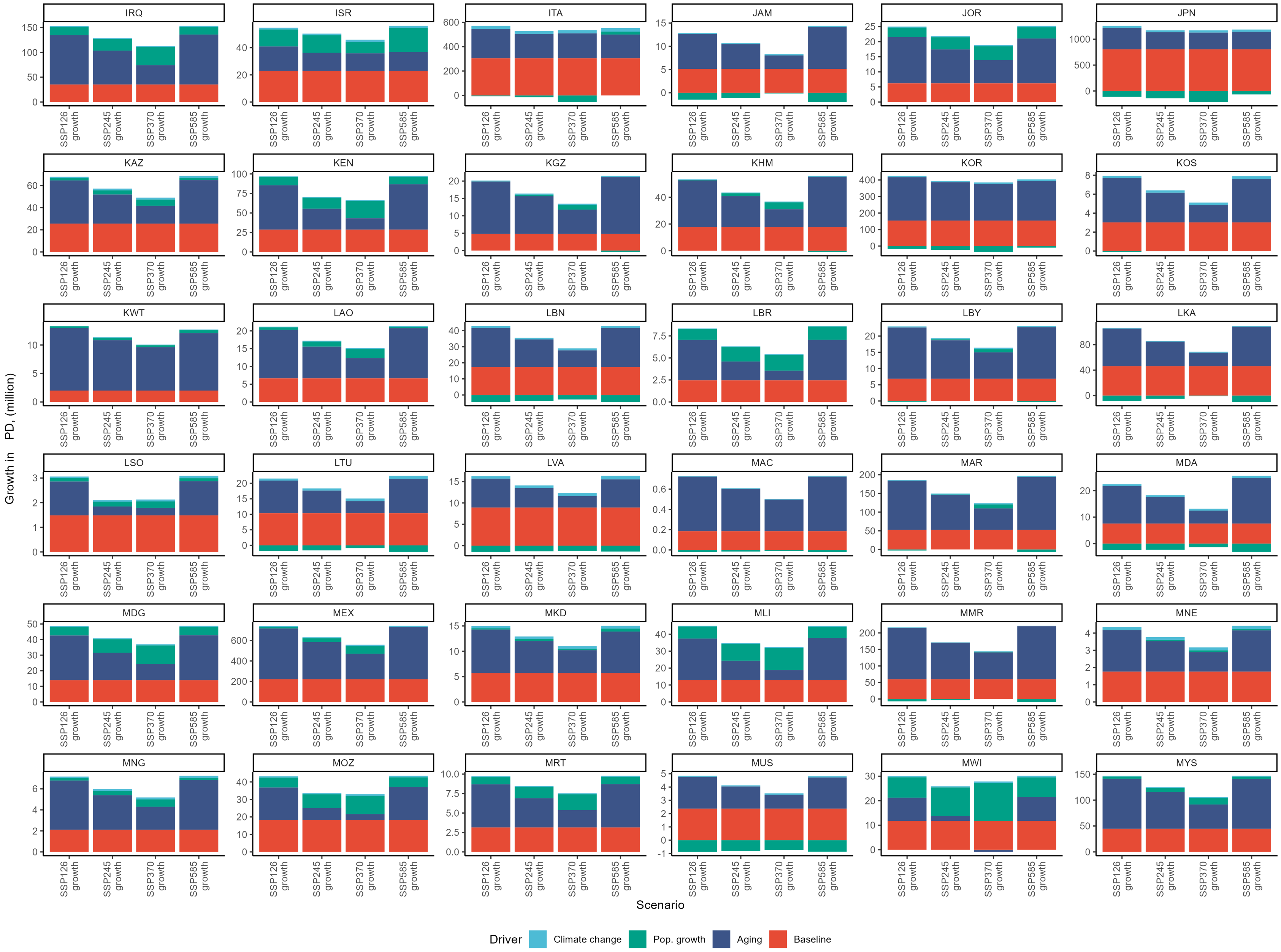

Figure SI-30. Country-level decomposition of determinants of $TMAX_{95}^{th}$ exposure E_r projections, by region and scenario.

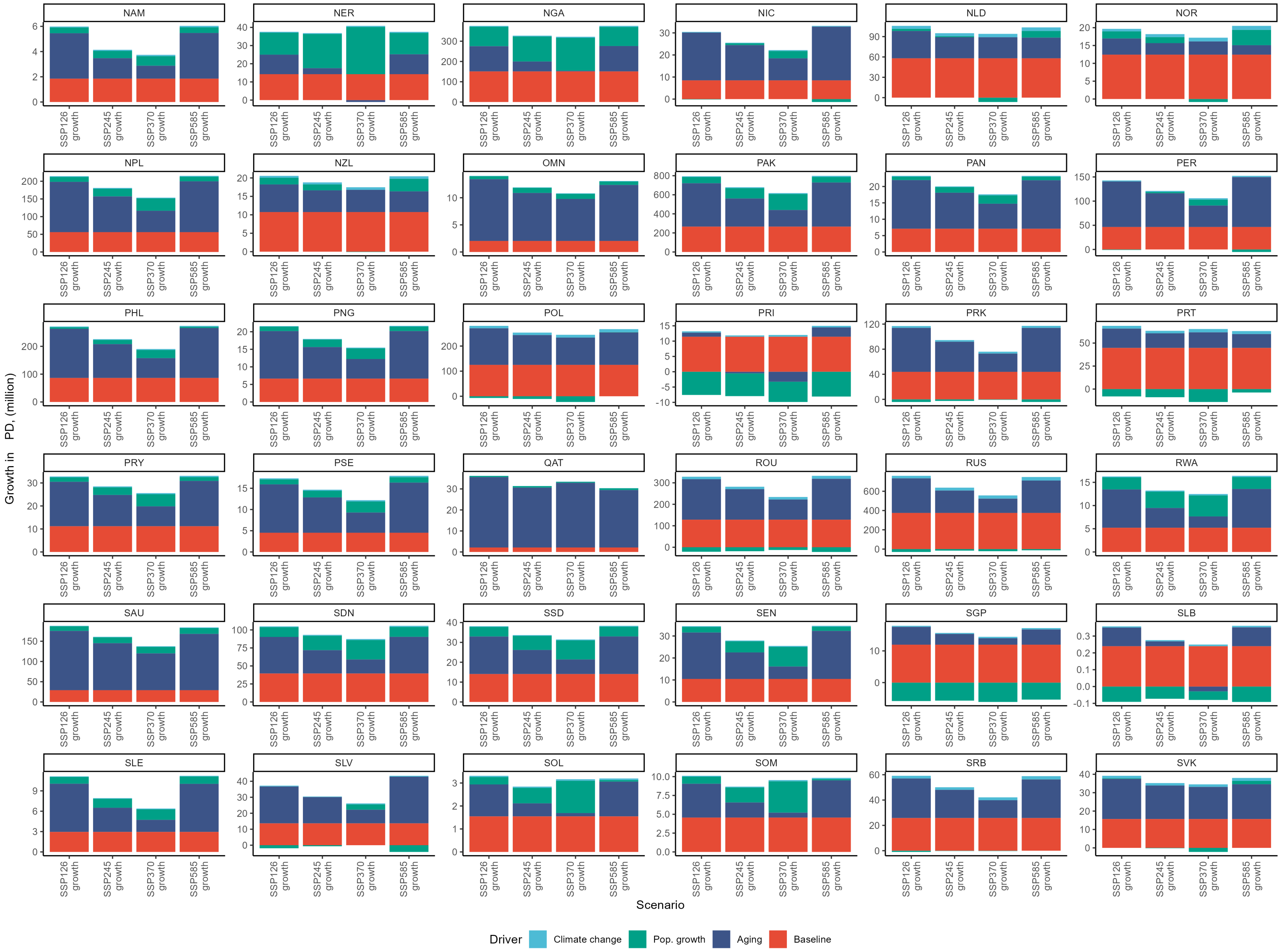

Figure SI-31. Country-level decomposition of determinants of $TMAX_{95}^{th}$ exposure E_r projections, by region and scenario.

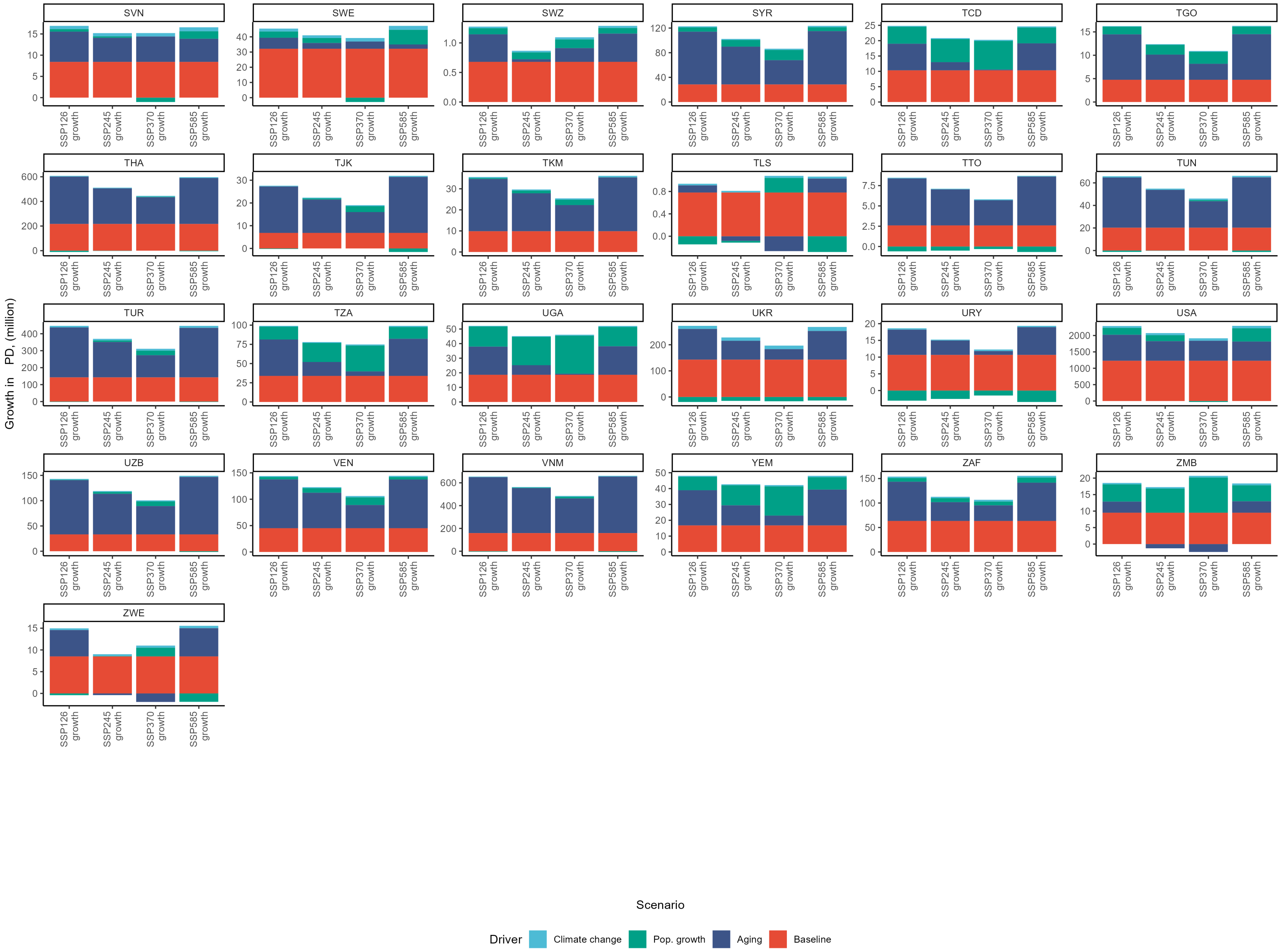

Figure SI-32. Country-level decomposition of determinants of $TMAX_{95}^{th}$ exposure E_r projections, by region and scenario.

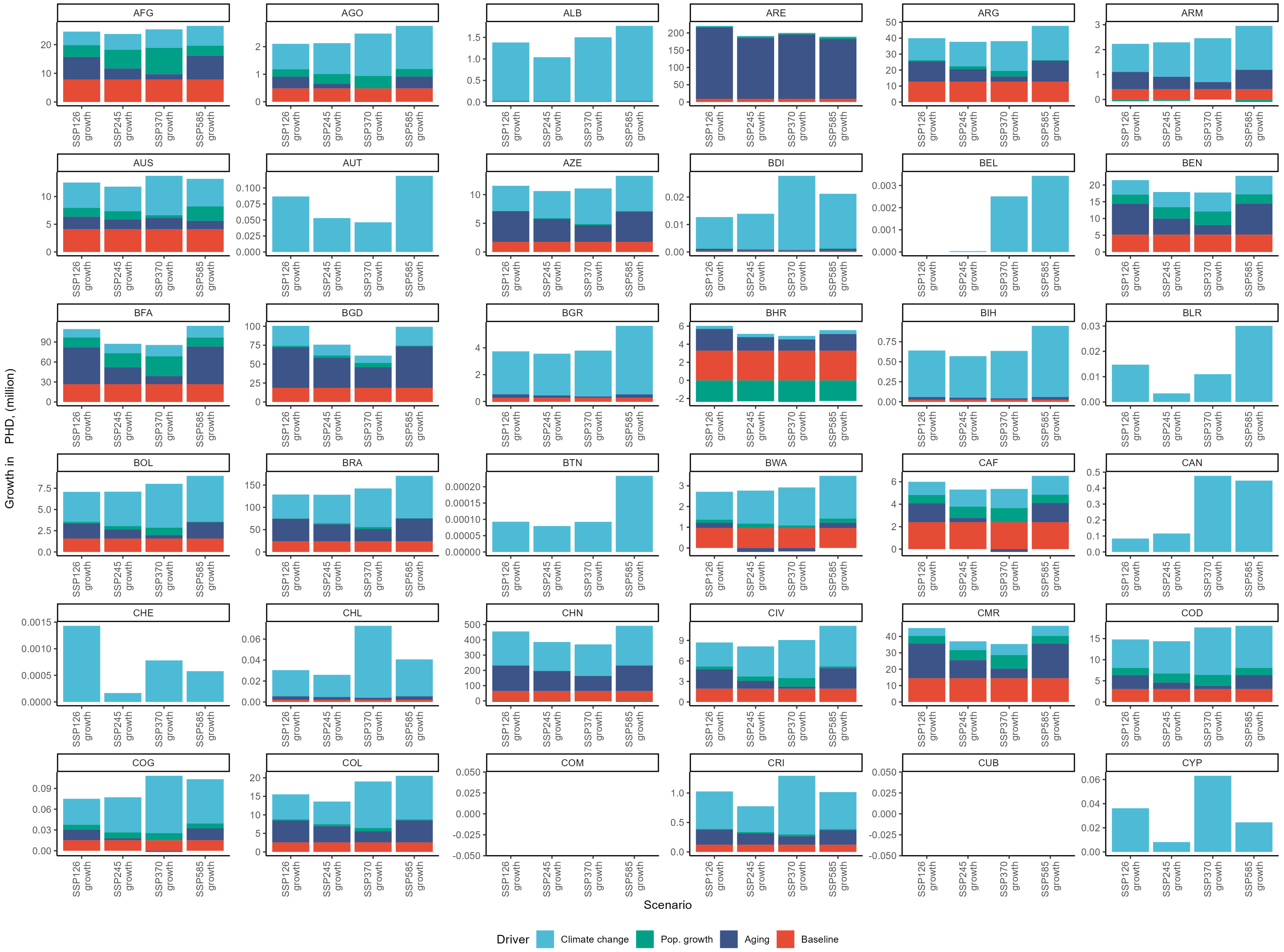

Figure SI-33. Country-level decomposition of determinants of #*hotdays* exposure E_r projections, by region and scenario.

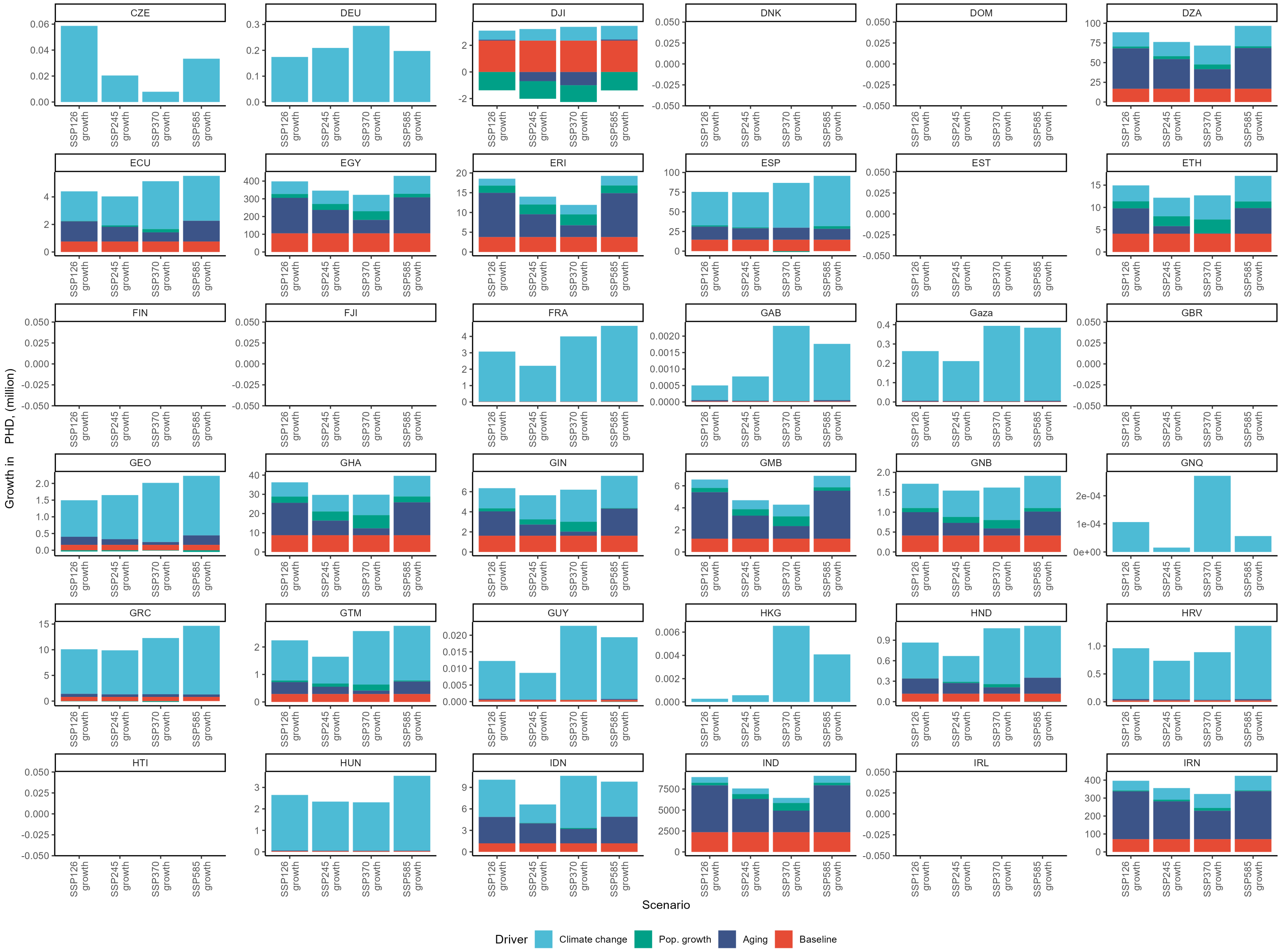

Figure SI-34. Country-level decomposition of determinants of #*hotdays* exposure E_r projections, by region and scenario.

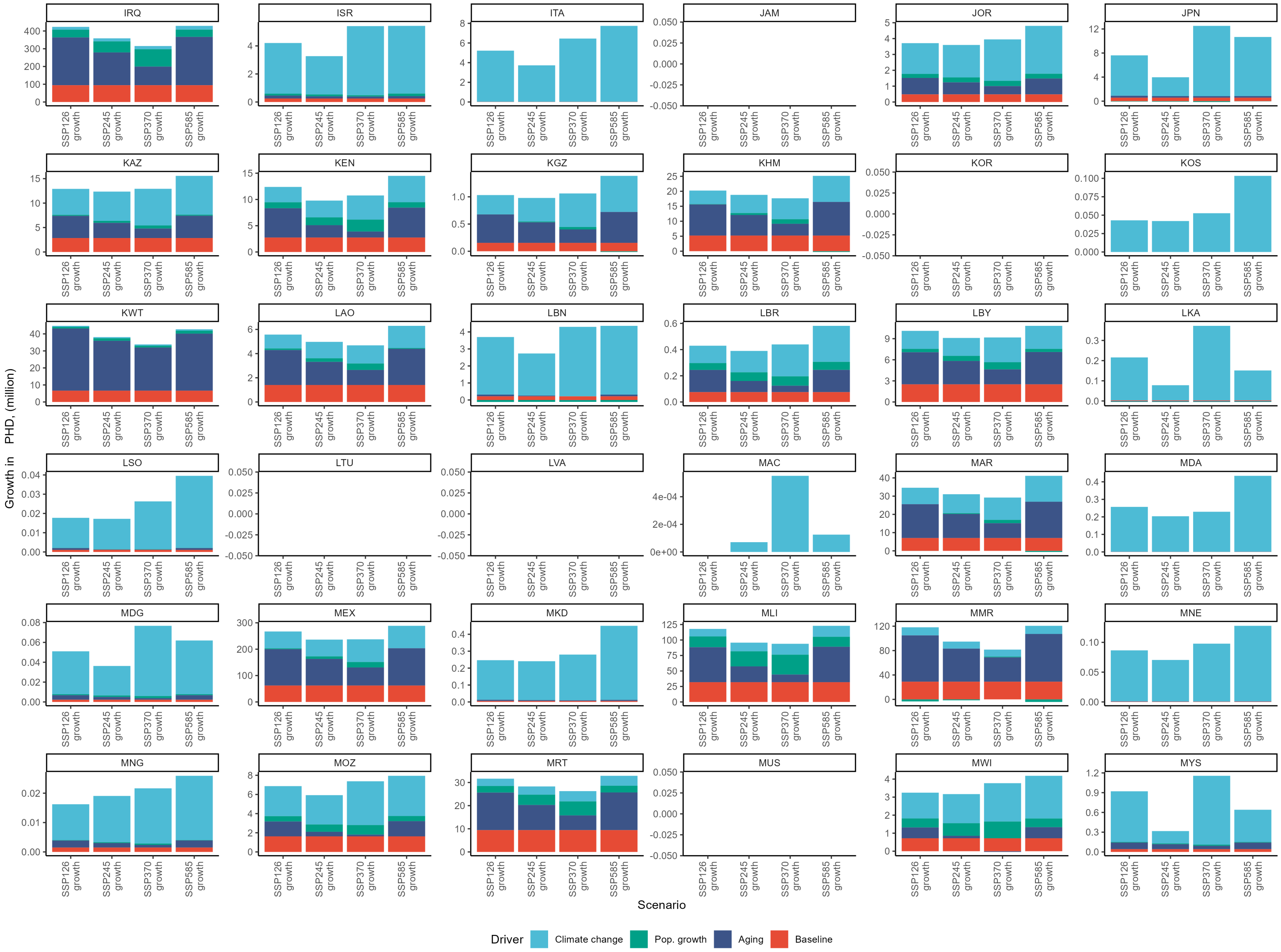

Figure SI-35. Country-level decomposition of determinants of *#hotdays* exposure E_r projections, by region and scenario.

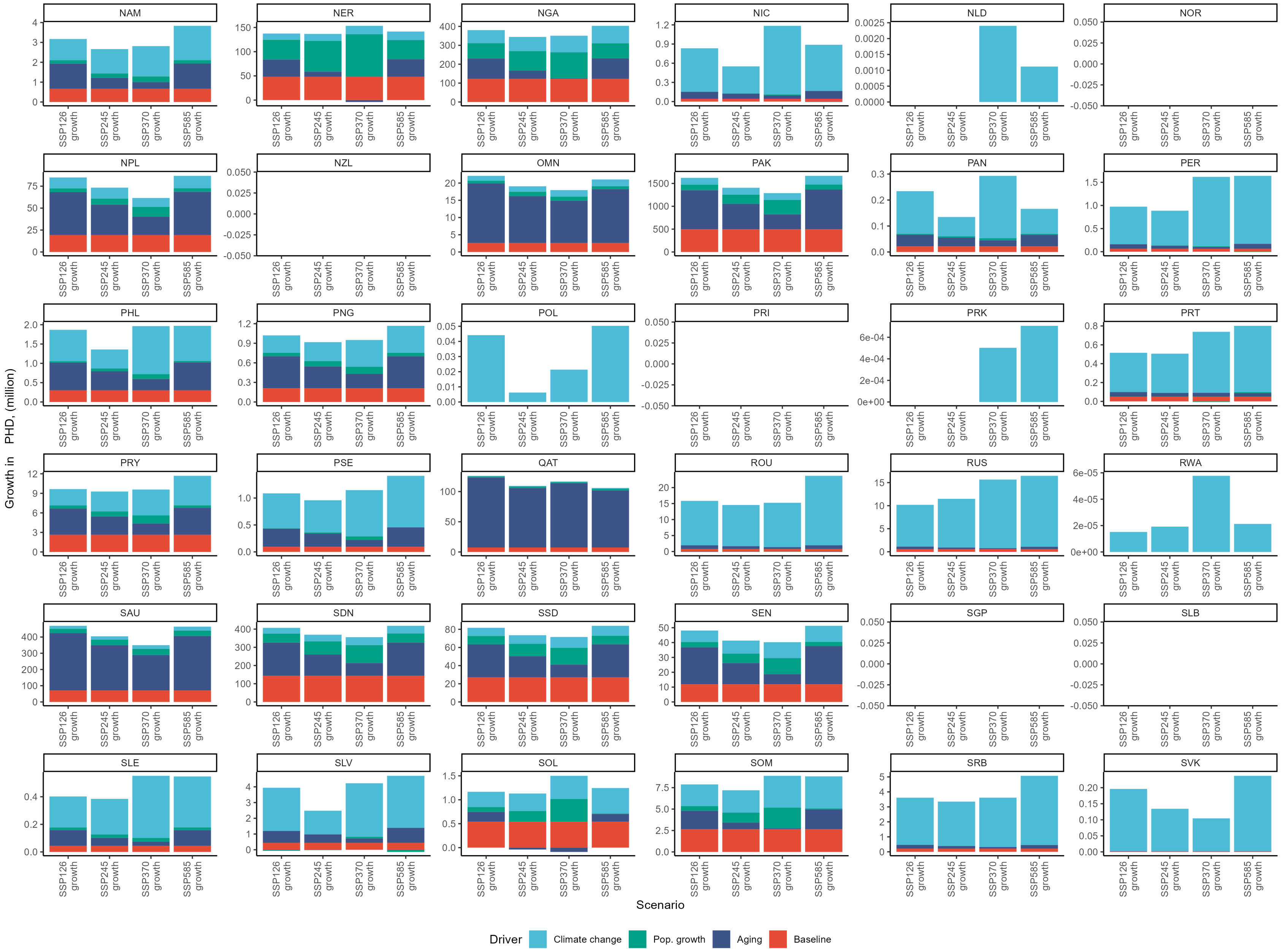

Figure SI-36. Country-level decomposition of determinants of $\#hotdays$ exposure E_r projections, by region and scenario.

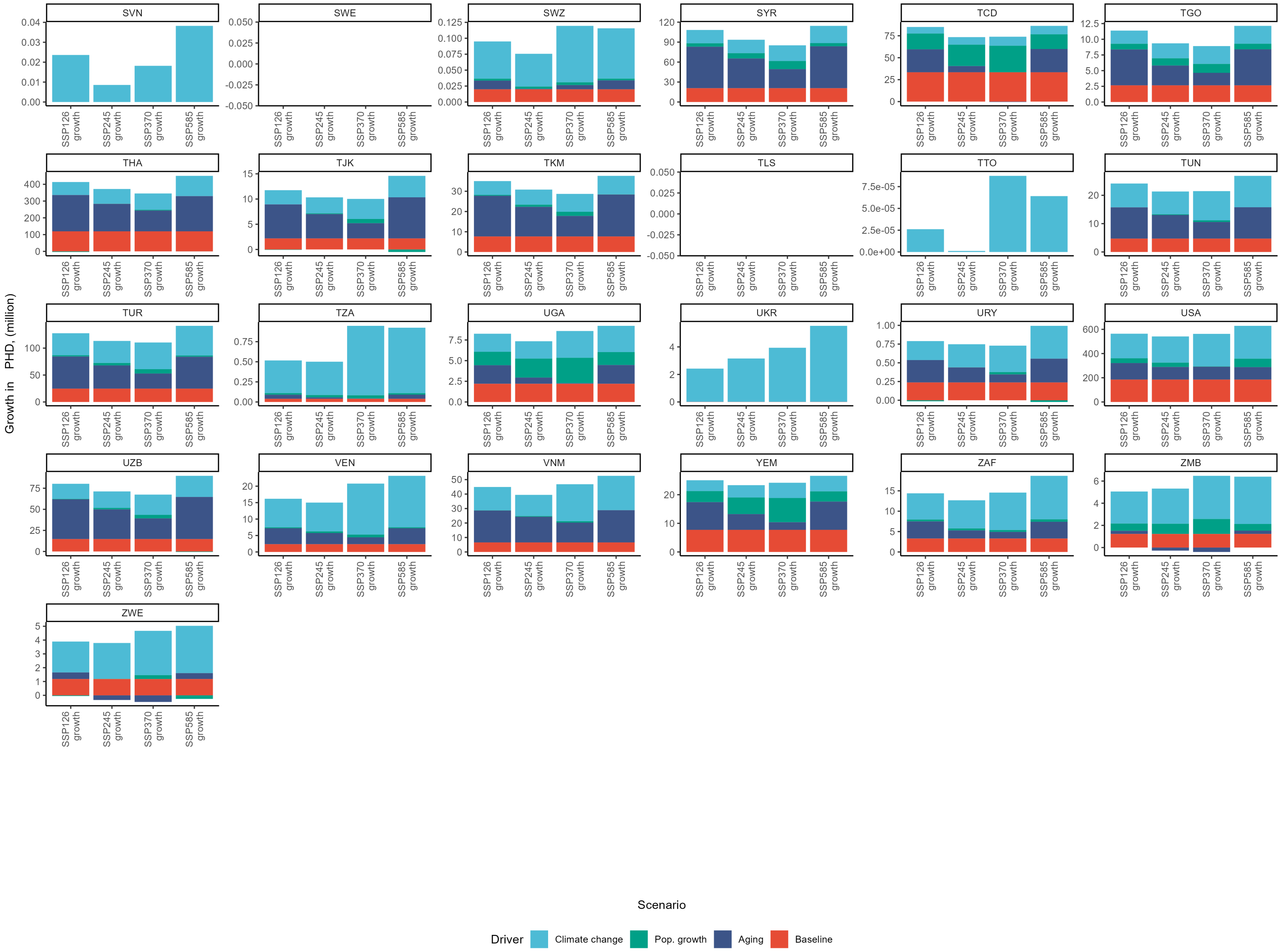

Figure SI-37. Country-level decomposition of determinants of #*hotdays* exposure E_r projections, by region and scenario.

Conceptual example of the R code implementation of age-stratified, grid cell-level down-scaled population projection

```r
# for reproducibility
set.seed(2023)

# like WorldPop, gridded, age-stratified population data (2020)
pop_grid_tot <- matrix(runif(100, min=0, max=50), nrow=10, ncol=10)
shares_grids <- sample(rnorm(100, mean=0.3, sd=0.05), 100, replace = T)
pop_grid_old <- pop_grid_tot * (1 - shares_grids)
pop_grid_young <- pop_grid_tot * shares_grids

# plotting and consistency check of WorldPop
sum(pop_grid_tot)
image(pop_grid_tot)
sum(pop_grid_old)
sum(pop_grid_young)
sum(pop_grid_old) + sum(pop_grid_young) == sum(pop_grid_tot)
image(pop_grid_old)
image(pop_grid_young)

# check current shares by age group
sum(pop_grid_young) / sum(pop_grid_tot)
sum(pop_grid_old) / sum(pop_grid_tot)

# like KC-Lutz, future country-level, age-stratified, pop. shares projections
age_str <- data.frame(year=rep(c(2010, 2050), each=2), gr=c("young", "old"), shares=c(0.3, 0.7, 0.25, 0.75))

# like Gao, future gridded population data (2050)
growth_rates <- sample(seq(0.15, 0.4, by=0.05), 100, replace = T)
pop_grid_tot_2050 <- pop_grid_tot * (1+growth_rates)
sum(pop_grid_tot_2050)
image(pop_grid_tot_2050)

###

# project downscaled pop. data (WorldPop) to be consistent with with KC-Lutz shares by age group

gr_rate_young <- (sum(pop_grid_tot_2050) * age_str$shares[age_str$year==2050 & age_str$gr=="young"]) /
(sum(pop_grid_tot) * age_str$shares[age_str$year==2010 & age_str$gr=="young"])

pop_grid_young_2050 <- pop_grid_young * gr_rate_young

gr_rate_old <- (sum(pop_grid_tot_2050) * age_str$shares[age_str$year==2050 & age_str$gr=="old"]) /
(sum(pop_grid_tot) * age_str$shares[age_str$year==2010 & age_str$gr=="old"])

pop_grid_old_2050 <- pop_grid_old * gr_rate_old

# check current counts of 2050 age-stratified population
sum(pop_grid_young_2050)
sum(pop_grid_old_2050)

# check discrepancy with Gao value
sum(pop_grid_young_2050) + sum(pop_grid_old_2050)
sum(pop_grid_tot_2050)

# re-calibrate age-stratified, gridded pop. projections to match total pop. gridded projections from Gao
delta <- sum(pop_grid_tot_2050) / (sum(pop_grid_young_2050) + sum(pop_grid_old_2050))

pop_grid_young_2050 <- pop_grid_young_2050 * delta
pop_grid_old_2050 <- pop_grid_old_2050 * delta

# check consistency with both Gao and KC-Lutz
sum(pop_grid_young_2050) + sum(pop_grid_old_2050)
sum(pop_grid_tot_2050)

sum(pop_grid_young_2050) / sum(pop_grid_tot_2050)
sum(pop_grid_old_2050) / sum(pop_grid_tot_2050)
```
